# Nano bio-robots: a new frontier in targeted therapeutic delivery

**DOI:** 10.3389/frobt.2025.1639445

**Published:** 2025-11-06

**Authors:** Mariya Anto, Koena Mukherjee, Koel Mukherjee

**Affiliations:** 1 Department of Bioengineering and Biotechnology, Birla Institute of Technology Mesra Ranchi Jharkhand, Ranchi, India; 2 Department of EIE, National Institute of Technology Silchar, Assam, India

**Keywords:** targeted drug delivery, bio-robots, actuation, therapy, drug release

## Abstract

In macro world, the robots are programmed machines, engineered to perform repetitive and often specialized tasks. Scaling down the size of a robot by a billionth of a meter gives a nano robot. The primary driving force behind the advent of micro and nano robots have always been the domain of medical technology and these robots are popularly known as nano bio-robots. The current review shines a light on these bio-robots in all their facets, encompassing both top-down and bottom-up fabrication approaches to their associated challenges followed by ethical approvals. The study describes in detail the synthesis techniques of nano bio-robot along with required actuation mechanism of the bio-robots. Further, in this paper, how a nano biorobotic drug-delivery system (NDDS) can deliver the drugs in a controlled way to the targeted site of the host in contrast to conventional drug administration is discussed. The paper also reviews and summarizes the administration pathways of these bio-robots in the human body and their efficacy in reducing various disorders. Overall, it can be said that the integration of nano robots with bio-concept presents distinct advantages and possesses significant promises for many applications.

## Graphical Abstract

**Figure:**
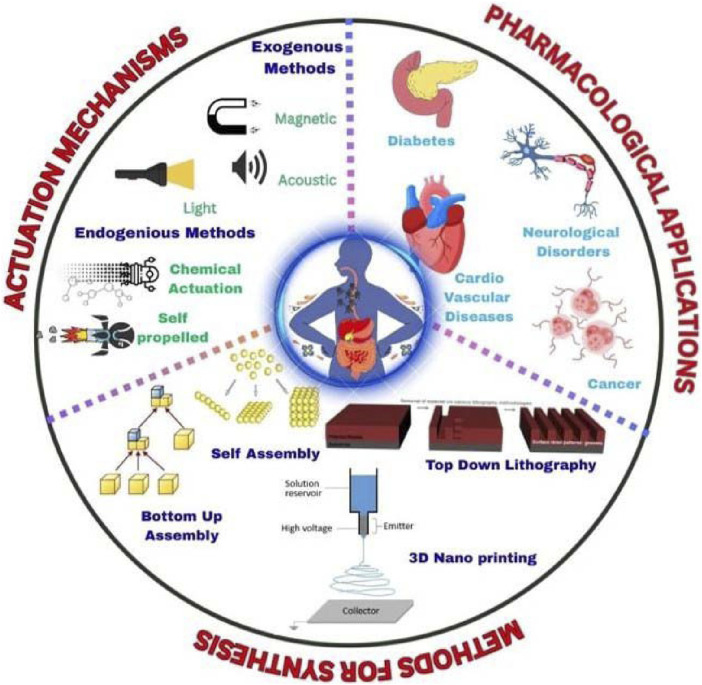

## Highlights

- Nano bio-robots minimize invasive surgery and real time diagnostic.
- Bio-robots with nanoscale functionality.
- Extensive research on long term effects in human body – major hurdle.
- Regulation in usage and discussion on risk factors.

## 1 Introduction

Nano sized biologically compatible robots called nano bio-robots or nano bio-robots are machines between 1 and 100 nm in dimensions and engineered to function at a scale that facilitates direct interaction within cells (Hulla et al., 2015). Vadlamani (2010) explains that these tiny robots can be programmed to interact with their environment on a molecular or cellular level and perform a variety of tasks. In light of their accuracy in manipulating chemicals, nano bio-robots have potential across various sectors, particularly healthcare, environmental monitoring, and manufacturing (Zhao et al., 2023). However, the concept of nano bio-robots revolves around utilizing advanced technology to manipulate materials at tiny scales with supramolecular dimensions where the process need to be operated at atomic and molecular scales (Wang, 2018). Autonomous robots at miniature scales exist to assemble themselves from basic components. The assembly process is controlled by intermolecular forces including hydrogen bonds and van der Waals interactions and hydrophobic effects (Lombardo et al., 2020). The process of autonomous assembly enables the creation of complex nanostructures which form the basis for nano bio-robots (Wei et al., 2021). Often times researchers have also focused on creating DNA based nano bio-robots which are called DNA Nanomachines where DNA is the body and DNA based computation serves as the brain. DNA origami is encapsulated with Iron (Fe) nanoparticles for external magnetic based actuation and mixed with drug. The actuation of nano bio-robot can be triggered using pH, temperature of the body. The development of nanotechnology enabled the construction of these robots more which show promise to revolutionize medical diagnostics procedures and drug delivery systems and surgical methods (Nelson et al., 2010; Klocke and Gesang, 2003).

**FIGURE 1:**
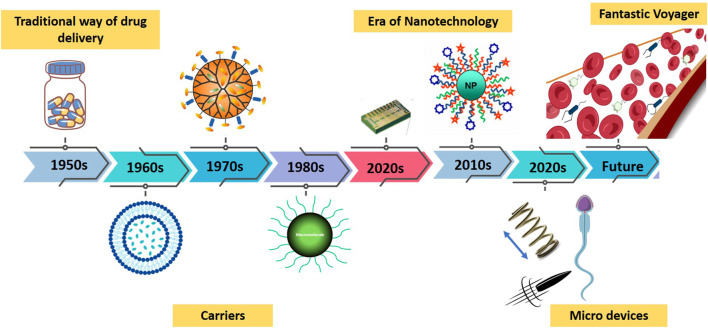
A timeline study of drug delivery approaches in human body.

Nowadays targeted drug delivery offers precision, efficiency and less toxic effects of drugs that are modified and fabricated for specific cells, tissues or organs (Vasantha Ramachandran et al., 2021). However, drug delivery path itself poses a long history (Figure 1) starting from 1950s when capsule system was in the market but after the oral administration, drug would release and sustain for 12 h only (Park et al., 2022). Gradually with time around 1980, the sustainability increased to 24 h. After that in late 1980s the first injectable and implants started their role in drug delivery for long month release. At the same timeline after the discovery of dendrimer structure, drug-polymer complexes were also introduced. In 1990 the first PEGylation concept came and after 2000 nanotechnology took control on drug delivery process with a broader application of targeted medicine. With the advancement of techniques for various nanomaterial fabrication (Allen and Cullis, 2013) and surface modifications, hydrogels (Lee and Mooney, 2001),nano fibers, SPIONs (Kandasamy and Maity, 2015; Kandasamy et al., 2018),quantum dots (Wegner and Hildebrandt, 2015), magnetic beads (Hu et al., 2018) show a promising path for targeted and nontoxic drug delivery. These drug delivery platforms act in a wide range from therapeutics to theranostics. Finally, greet acceleration came in the field of targeted drug delivery when first the concept of micro/nano robots came. Integration of nanotechnology, electric engineering, mechanical engineering, control engineering, chemical engineering and biotechnology provide the advantages and distinctive benefits of robot in precise drug transport and delivery. These micro/nano robots adeptly tackle the difficulties faced by conventional robots in domains like as navigation, perception, cognition, and other facets, particularly in the context of cancer and cardiovascular disorders (Cao et al., 2024). These micro/nano robots solved the problems of complex biological system by a mix approach of localization and navigation in the microenvironment using real time monitoring. The concept of “fantastic voyager” was conceived (Feynman, 2012; Fleischer, 1966) which refers to a micro robot that is capable of being remotely operated within the human body in order to conduct tests, administer payloads, and perform surgical procedures if needed (Ramachandran et al., 2021).

Similar to any macro world robotic system, a nano bio-robot also require energy supply to achieve mobility and perform manipulation. Nano bio-robots receive their power through various methods which include chemical reactions as well as external magnetic fields and light (phototaxis) and ultrasound. The power source selection strongly depends on the robot’s function together with its operational environment. Nano bio-robots controlled by magnets show exceptional value in healthcare due to their ability to receive remote guidance during body procedures (Wang et al., 2021) and well established MRI technology further aids in the nano bio-robot control. However, precise drug delivery necessitates use of nano sensors which enable them to detect triggers including pH levels and temperature and magnetic fields. The sensors guide the robot through its environment and the actuators enable the robot to execute operations such as nanoparticle manipulation and surgical procedures.

The focus of developing a nano bio-robot is mainly for precise targeted drug delivery at nanoscale which require precise actuation. However, *in vivo* actuation at nanoscale is often limited by the dominance of Brownian Motion due to random thermal motion of molecules and it is stronger than any applied force. This makes controlled actuation very hard and challenging for any nano bio-robot. Moreover, presence of viscous forces is dominant in blood vessel, due to which conventional propulsion techniques are not much effective. To create adequate actuation force, several techniques like molecular motors, magnetic particles, DNA origami devices are used but the magnitude of generated force are minuscule. Furthermore, nano bio-robots do not carry onboard power source and use of external power source such as magnetic field, light, acoustic waves limit autonomy of nano bio-robot and workspace of the robot. Another difficulty related to action of nano bio-robots are precise timing of drug release as sensor signals such as pH, temperature change is often of very small magnitude. In addition to that, medical applications require nano bio-robots to use materials which prevent immune reactions in the human body to avoid adverse immune responses. These materials often include polymers, liposomes structures and metallic nanoparticles covered with substances compatible, with the immune system (Cavalcanti et al., 2008a).

This current review provides an extensive summary of recent studies about micro and nano bio-robots used for drug delivery applications. Our research will examine the materials and production methods used to develop these robots together with their operational mechanisms and targeted medical applications where they show remarkable promise. Through exploring the progress and obstacles, in this quickly developing area of study we hope to showcase the possibilities of micro and nano bio-robots in revolutionizing drug delivery practices and opening doors for enhanced and tailored medical care solutions.

## 2 History of nano bio-robots

The evolution of nano bio-robot technology from ponderings to real world applications has unfolded over numerous decades and is deeply connected to the forward-thinking concepts of early scientists. The inception of nano bio-robots can be linked back to the ideas put forth by physicist Richard Feynman. In 1959, Feynman presented a speech entitled “There’s Plenty of Room, at the Bottom” during an American Physical Society gathering. In this address he introduced the notion of manipulating individual atoms to create incredibly minuscule machines designed for specific functions. Feynman had a vision of a future where tiny machines, which he compared to “scalpels and forceps”, could construct structures atom by atom. While his concepts were speculative then, they set the groundwork for the development of nanotechnology and nano bio-robotics in the future (Tsuda, 2016).

The advancement of nano bio-robots saw progress during the 1980s and 1990s due to the contributions of K. Eric Drexler. The pioneer of nanotechnology Drexler built upon Feynman’s concepts and introduced the notion of molecular nanotechnology, in his influential book “Engines of Creation” (Drexler, 1987). Drexler proposed the development of “assemblers”—imaginary nanomachines capable of manipulating individual atoms to construct various structures (Figure 1). Drexler’s concept of assemblers has the potential to unlock uses ranging from molecular production to cutting edge medical treatments. His groundbreaking research played a role, in shaping nanotechnology into a well-defined scientific field and motivated a fresh wave of scientists to delve into the capabilities of nano bio-robots.

One significant breakthrough in this field involved creating robots at the micro and nanoscale for medical purposes (Sun et al., 2024). They were intended to carry out different functions like delivering medication to specific areas efficiently by transporting healing substances right to affected cells to reduce side effects and enhance treatment effectiveness Additionally nano bio-robots were studied for their possible use, in precise surgeries allowing them to move through the body to conduct intricate surgical operations with exceptional precision Another exciting use of nano bio-robots is their potential to cleanse the blood by eliminating toxins. The research demonstrates their potential to treat conditions such as sepsis and heavy metal poisoning (Li J. et al., 2017).

Despite the achievements in nano bio-robotics field and its progress over time, a major hurdle faced is the control and maneuverability of these minuscule devices within the intricate landscapes of the human body or other operational scenarios (Martel, 2014). Traditional control techniques often fell short when it came to manipulating nano bio-robots with the precision, for practical purposes. This obstacle was tackled by utilizing fields as an external source of activation (Martel et al., 2008). This allowed for handling, reconfiguration and customization of micro and nano bio-robots. In addition to magnetic manipulation capabilities various technological progressions have played a role in the development of nano bio-robots. For instance, scientists have delved into imitating nature when creating nano bio-robots that emulate the actions of living organisms like bacteria or cells. This bio inspired nano bio-robots are typically tailored with functions, such as navigating through natural obstacles such as the blood brain barrier, which holds important implications.

## 3 Synthesis of nano bio-robots

Creating nano bio-robots involves a multi-faceted process that combines different tiny components to form operational systems at the nanoscale level. There are two methods used for producing nano bio-robots; top down and bottom-up strategies, each with their own strengths and weaknesses. These methods allow for the development of nanostructures, with customized characteristics suited for particular uses (Table 1).

**TABLE 1:** Comparison of various methods used in synthesis of nanorobots.

Fabrication Paradigm	Method	Resolution	Scalability	Material Versatility	Key Application Examples	Critical Challenges
Top-Down	Photolithography	Sub-micron to a few micrometers (Li et al., 2017a)	Mass Production (Li et al., 2017a)	Photoresists, metals, silicon (Weerarathna et al., 2025)	Self-folding microgrippers, microfluidic devices (Li et al., 2017a)	Resolution limits, mask requirement
Top-Down	Electron-Beam Lithography (EBL)	Sub-10 nm (Prizes, 2020)	Prototyping, Lab-Scale (Karimi et al., 2024)	Polymers, metals (Weerarathna et al., 2025)	NEMS actuators, quantum sensors, photonic circuits (Lee, 2022)	Slow speed, high cost, low throughput (Karimi et al., 2024)
Top-Down	Focused Ion Beam (FIB) Milling	<50 nm (Focused Ion Beam, n.d.)	Prototyping, Lab-Scale (Buchnev et al., 2022)	Metals, semiconductors, dielectrics (Buchnev et al., 2022)	Nanosculpting, thermally actuated grippers (Benouhiba et al., 2021)	Complex optimization, slow throughput (Buchnev et al., 2022)
Bottom-Up	Chemical Vapor Deposition (CVD)	Atomic layer control	Industrial scale (Escorcia-Díaz et al., 2023)	Carbon, silicon, dielectrics (Micro/nano fabrication, n.d.)	Carbon nanotubes, electronic components (Cavalcanti, 2023)	Requires high temperatures, specific precursors (Cavalcanti, 2023)
Bottom-Up	Molecular Beam Epitaxy (MBE)	Atomic layer control	Industrial scale (McCray, 2007)	III-V semiconductors, metals (McCray, 2007)	Transistors, laser diodes, quantum dots (McCray, 2007)	Ultra-high vacuum, high cost (McCray, 2007)
Bottom-Up	DNA Origami	Sub-100 nm (Lu et al., 2019)	Lab-Scale, parallel synthesis (Lu et al., 2019)	DNA strands, biomolecules (Lu et al., 2019)	Targeted cancer therapy, molecular machines (Lu et al., 2019)	Biocompatibility, payload stability, actuation (Lu et al., 2019)
Bottom-Up	Two-Photon Polymerization (TPP)	Sub-micron (Huang et al., 2020)	Prototyping, Lab-Scale (Huang et al., 2020)	Photopolymer resins (Huang et al., 2020)	3D microgrippers, helical microswimmers (Huang et al., 2020)	Material limitations, printing time for complex shapes (Huang et al., 2020)
Bottom-Up	Electrospinning	Tens of nm to μm (People, 2023)	Mass Production (Ju et al., 2025)	Polymers like polycaprolactone, Poly (lactic-co-glycolic acid), biopolymer composite (Ju et al., 2025)	Nanofiber bodies, drug delivery scaffolds (Castillo-Henríquez et al., 2020)	Requires post-processing for propulsion (Ju et al., 2025)

### 3.1 Top-down lithography

Top down lithography is a technique used in manufacturing processes, creating structures from larger bulk materials, through top down lithography techniques can be photolithography, Electron-beam lithography (EBL) as well, as focused ion beam (FIB) milling.

#### 3.1.1 Photolithography

Photolithography is a known method in the field of microelectronics for creating patterns on a surface material such as a substrate. The working principle of this method is that, a sensitive substance called a photoresist is applied onto the substrate and then exposing it to ultraviolet light using a mask with a specific pattern. The sections of the photoresist that are exposed, and undergo chemical changes enabling them to be taken off selectively in order to form the intended design, on the surface material. Photolithography is quite useful in producing micron details; however it faces limitations due, to the diffraction constraints of light when dealing with structures below 100 nm (Sharma et al., 2022).

- Advantages: The advantages of this method include its ability to process large volumes at high resolution (extreme UV lithography achieves resolutions down to tens of nanometers).
- Limitations: The technology faces restrictions from diffraction limitations which become significant when wavelengths drop below 100 nm and it demands costly equipment and specialized environmental controls while performing poorly with three-dimensional (3D) nanostructure formation.

#### 3.1.2 Electron-beam lithography (EBL)

Electron-Beam Lithography involves using an electron beam to write designs directly onto an electron sensitive resist material with high precision and detail accuracy than traditional photolithography methods can achieve. It is commonly employed for producing nanostructures with minute features measuring a few nanometers in size. EBL is especially valuable for crafting accurately shaped nano bio-robots despite being a time consuming and costly procedure due to the step, by step electron beam scanning involved (Chen and Dai, 2018).

- Advantages: The process delivers nanostructures with high resolution capabilities down to 2 nm while operating without masks to benefit research and prototyping activities and creating complex detailed nanostructures.
- Limitations: The process operates at a slow pace while being expensive to maintain and it fails to work well with large-scale manufacturing needs.

#### 3.1.3 Focused ion beam (FIB) milling

FIB milling is a process where a material is targeted with an ion beam made up of gallium ions to carve away material and form nanoscale structures accurately and is commonly employed for prototyping and adjusting nano bio-robots; however it may lead to material damage, from the high energy ion impacts that could restrict its application in delicate biological contexts (Nelson et al., 2010; Nebraska Center for Materials & Nanoscience, 2012).

- Advantages: The direct-write technique enables fast modifications and high spatial precision down to 5 nm while allowing processing of different materials including metals semiconductors and polymers.
- Limitations: The process suffers from material damage caused by gallium ions yet it operates slowly and costs high operational expenses and lacks scalability for mass production.

### 3.2 Bottom-up assembly

The construction of nano bio-robots through bottom up assembly requires the precise assembly of these small machines from individual atoms or molecules by mimicking the self-assembly processes of biological systems to construct complex structures piece by piece.

#### 3.2.1 Chemical vapor deposition (CVD)

Chemical vapor deposition is widely used in producing nanomaterials like carbon nanotubes and graphene that play roles in various nano bio-robots functionalities. In the CVD process volatile precursor chemicals are introduced into a reaction chamber where they break down and create a film layer over a substrate to form the desired nanostructure. This method offers manipulation over material thickness, composition and crystallinity making it a crucial approach, for crafting nano bio-robots (Sitti, 2009).

- Advantages: The process demonstrates scalability while producing nanomaterials across extensive areas and it enables uniform materials with high purity and allows modification of material properties for nano bio-robot functionality.
- Limitations: The process demands high temperatures which restricts material compatibility and handling toxic precursor gases while the deposition rate remains slower than other thin-film fabrication methods.

#### 3.2.2 Molecular beam epitaxy (MBE)

In MBE atoms or molecules are precisely directed onto a surface in a vacuum environment to create thin and even layers of material with great accuracy, at the atomic level. MBEp is commonly employed in producing semiconductor nanostructures that can be utilized in nano bio-robots for a range of optical purposes (Asahi and Horikoshi, 2019).

- Advantages: Atomic-level precision during the process enables highly controlled nanostructures and high-purity deposition which reduces material defects while making heterostructures suitable for material combination
- Limitations: The process operates at an extremely slow pace which prevents its use in mass production and requires ultra-high vacuum systems that drive up costs while remaining sensitive to contaminants that need strict environmental controls.

#### 3.2.3 DNA origami

**FIGURE 2:**
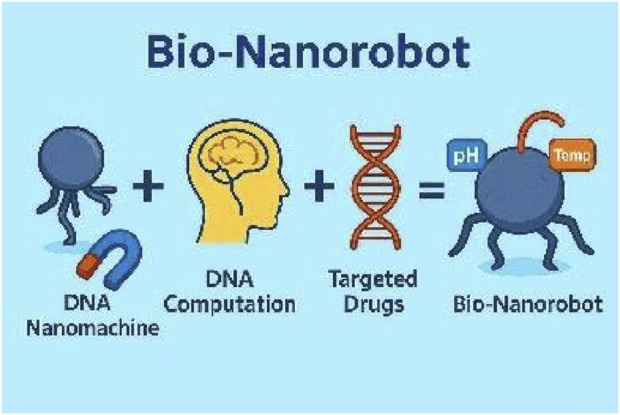
DNA nanomachines - a nano bio-robot made of DNA origami, magnetic, pH and temperature based actuation.

DNA Origami (Figure 2) is a technique that involves folding single stranded DNA molecules into precise shapes using complementary base pairing from the bottom up methodically revolutionizing nanotechnology applications like creating nano bio-robots with customizable structures and functionalities, for targeted drug delivery or molecular sensing purposes (Cavalcanti et al., 2008a).

- Advantages: The system demonstrates both self-assembly capabilities and programmability while being biocompatible and mass producible through biological methods.
- Limitations: The structural stability of these systems tends to fail when exposed to harsh environmental conditions and their mechanical strength limitations restrict particular uses and the fabrication process demands time-consuming strand design and assembly.

### 3.3 Three dimensional nanoprinting

Nanotechnology has advanced with the introduction of nano printing technology that facilitates the development of nanobots with complex shapes and capabilities not achievable, through conventional lithography techniques limited to two dimensional or quasi two dimensional designs (Chen et al., 2020).

#### 3.3.1 Two-photon polymerization (TPP)

Two-Photon Polymerization is a method in printing that involves utilizing a concentrated laser beam to solidify a light sensitive resin at a specific point of focus while adjusting this focal point across three dimensions to create intricate nanostructures layer by layer with excellent sub micron precision resolution ideal for producing nano bio-robots equipped with detailed features essential, for various biomedical uses (Nelson et al., 2010).

- Advantages: Sub-micron resolution (∼100 nm), true 3D fabrication unlike traditional lithographic techniques and compatible with biocompatible and biodegradable materials for medical applications.
- Limitations: Slow fabrication speed that might limit scalability, requires expensive femtosecond lasers and precise control systems and material limitations, as only photosensitive polymers can be used.

#### 3.3.2 Electrospinning

Electrospinning involves a method used to create nanofibers that can be incorporated into three dimensional formations. These nanofibers are customizable with polymers to facilitate specific interactions with biological substances for applications, in drug delivery and tissue engineering purposes (Chen and Dai, 2018).

- Advantages: Simple and cost-effective compared to other nanoscale fabrication techniques, scalable and high-yield, making it ideal for large-scale production and customizable fiber morphology and surface chemistry, allowing for functionalization in biomedical applications
- Limitations: Requires careful solvent selection, limited structural control and mechanical properties may be weaker compared to bulk materials.

### 3.4 Self-assembly techniques

Self-assembling methods make use of the characteristics of molecules to autonomously arrange themselves into predetermined shapes or forms (Amadi et al., 2022). This method proves useful in the creation of nano bio-robots where meticulous management of the construction procedure becomes essential (Wei et al., 2021).

Supramolecular Chemistry is a field that utilizes covalent interactions like hydrogen bonding and van der Waals forces to construct nanostructures. These interactions play a role, in creating durable and efficient nano bio-robots capable of reacting to their surroundings. A quality that makes them well suited for drug delivery and biosensing applications (Wei et al., 2021). Using guidance in assembly involves the use of external magnetic fields to direct the positioning and motion of tiny magnetic particles that come together to form bigger structures. This approach is especially valuable for developing nano bio-robots that can be manipulated from a distance inside the body. Such as, in specialized drug delivery setups (Cavalcanti et al., 2008a).

However, the development of nanorobotics will not be depended on a single manufacturing approach. The evidence shows that future nanorobotics will use multiple fabrication techniques to benefit from each method’s unique capabilities. The structural body of a nanorobot can be produced through photolithography as a top-down method to create strong mass-produced features (Li J. et al., 2017). The body structure would receive its functional components from bottom-up synthesis of DNA origami “kill switches” for drug delivery applications (Högberg and Hammarskjöld, 2024). The combination of top-down structural methods with bottom-up functional techniques through this synergistic approach enables researchers to overcome the individual constraints of each paradigm.

Artificial intelligence (AI) and machine learning integration represents a significant transformative trend in fabrication processes (Buchnev et al., 2022). AI systems analyze fabrication data to optimize process parameters in real-time which accelerates research and development cycles (Buchnev et al., 2022). The capability decreases the need for prolonged trial-and-error procedures while enhancing result reproducibility and enabling complex process integration (Buchnev et al., 2022). The field is on the verge of a significant paradigm shift which will transform it from basic macro-scale machine miniaturization to the development of autonomous intelligent microscopic devices.

## 4 Nano bio-robot actuation and drug release

The integration of nanotechnology into medicine will revolutionise the way drugs are delivered to specific locations within the body in nano scale. Nano bio-robot actuation and drug release represent critical aspects of this innovation, focusing on how these microscopic machines move and how they control the release of therapeutic agents. Advanced actuation mechanisms enable precise navigation, while drug release mechanisms ensure targeted and efficient therapy with minimal side effects. Below, we delve into these concepts in detail.

### 4.1 Actuation mechanism

Efficient actuation mechanisms are required by the nano bio-robots to navigate through complex biological environments. The actuation mechanisms are engineered to provide control over the nano bio-robot’s position, speed, and direction, crucial for targeted drug delivery. In the absence of external energy source, actuation require to transform energy available to the nano bio-robots (Hu et al., 2020). Migration of these nano robots are based on different sources of magnetic field, sound waves, chemical synthesis, light origin, electric field and biological structures. Depending on the sources these robots are also classified such as magnetically/acoustic driven or powered robot. Various research suggests various names for these robots, nano-grippers, nano-drillers, nano-swimmers, nano-scavengers and many more (Zhou et al., 2021). However, the utilization of these robots is same, mainly for targeted drug delivery and precise surgery.

**FIGURE 3:**
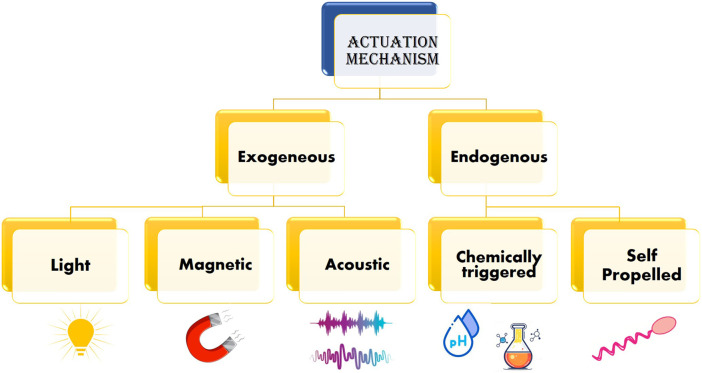
Various actuation mechanisms used by nano bio-robots.

The nano bio-robots must be administered into bloodstream to deliver the targeted drug (Martel, 2010). However, in order to do so, the drug along with the nano bio-robot must overcome the hydrodynamic drag due to viscoelastic properties of the blood itself (Ahmed et al., 2015). Therefore, based on the type of energy, key actuation methods are classified as shown in Figure 3.

**FIGURE 4:**
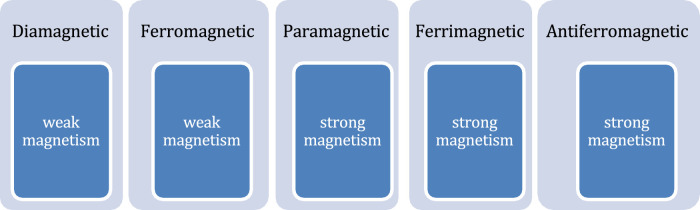
Types of magnetic materials used in magnetic nanorobots.

#### 4.1.1 Exogenous method

This method employs usage of external power driven nano bio-robots. Notable fields of research are magnetic field based, Visible and NIR spectrum of light energy and Acoustic energy. Although, magnetic field based nano bio-robot actuation has gained attention in the research community due to its non-invasive nature, minimum biological damage and well-established MRI technology in the medicine domain.

##### 4.1.1.1 Magnetic actuation

Magnetic fields are among the most commonly employed methods for actuating nano bio-robots due to their remote controllability in deep or hard-to-reach areas, such as the brain or thin blood vessels and biocompatibility. Iron (Fe) based nanoparticles with magnetic property are integrated into the nano bio-robots, allowing them to respond to external magnetic fields. The concept of actuation by magnetic field was initiated by Dreyfus et al. (Dreyfus et al., 2005) in the year 2005. Magnetic nanorobots in the presence of proper magnetic field can be remotely actuated in water, blood, and even cell tissue fluid.

##### 4.1.1.2 Magnetic materials used in MNRs

The choice of an external instrument to provide magnetic field for actuation of magnetic nanorobots (MNRs) depend on the type of magnetic particle used to develop the nano bio-robot. The magnetic materials (Table 2) exhibit distinct magnetic properties when subjected to magnetic fields. Furthermore, experiments were performed with various frequency of oscillating magnetic fields and magnetic field strengths in (Li et al., 2016; Li T. et al., 2017; Xu et al., 2021). According to the alignment and response of magnetic dipoles, the MNRs can be divided into different categories as shown in Figure 4.

**TABLE 2:** Various magnetic material used in nano bio-robot actuation mechanism.

Material	Magnetic Property	Biocompatibility	Advantages	Limitations	Applications
Iron Oxide (Fe_3_O_4_, γ-Fe_2_O_3_)	Ferromagnetic	Biocompatible (FDA-approved)	Safe, stable, MRI-compatible	Moderate magnetic strength	Drug delivery, imaging, biosensing
Cobalt (Co), Cobalt Ferrite (CoFe_2_O_4_)	Hard magnetic (high coercivity)	Toxic	Strong magnetic response	Toxicity, stability issues	Actuation in strong fields
Nickel (Ni)	Ferromagnetic	Toxic	Easy fabrication (nanowires, thin films)	Biotoxic, prone to corrosion	magnetic swimmers
Rare-Earth Magnets (NdFeB, SmCo)	Permanent, very high magnetic strength	Poor	High saturation magnetization	Expensive, brittle, low biocompatibility	High-force actuation
Magnetic Alloys (FePt, FeCo)	High magnetic saturation, stable	Moderate	Stronger actuation, stable in harsh environments	Costly, fabrication complexity	Precision control, biomedical actuation
Hybrid/Composite (e.g., Fe_3_O_4_ core + gold/silica shell)	Tunable (depends on core)	Good	Combines strong actuation + drug loading/functionalization	More complex fabrication	Multifunctional MNRs

In the presence of an external magnetic field, the initially disordered magnetic moments get reoriented and starts exhibiting paramagnetic nature. However, the other forms of magnetism (ferromagnetism, ferrimagnetism, or antiferromagnetism) are produced by the interaction of magnetic dipoles. A brief comparison chart is shown here to compare between different materials regarding their magnetic strength and biocompatibility for use in nano bio-robots.

According to recent progress in the field of MNRs, Iron Oxide based materials have ferromagnetic property and exhibit less toxicity thereby proving to be biocompatible according to Federal Drug Association (FDA). However, certain disadvantages of magnetic nanorobots are toxicity of materials, limited penetration depth of magnetic fields, possible tissue heating, long-term safety concerns and limited to patients with metal implants.

- Mechanism: By varying the strength and direction of a magnetic field, nano bio-robots can be guided to their target locations. Rotating magnetic fields induces helical or spinning motion in screw-shaped nano bio-robots, mimicking the motion of bacterial flagella for efficient navigation, thus enabling the nano bio-robot to propel through viscous fluids.
- Limitations: The magnetic actuation-based technique cannot be employed in people having metal implants like clips or artificial pacemakers in their bodies. Limitations of this technology along with possible future direction has been emphasized in (An et al., 2023) by Miao et al.

##### 4.1.1.3 Acoustic actuation

An acoustic wave is a mechanical wave generated using a transducer (usually a piezoelectric transducer). The vibrations of piezoelectric transducer creates an acoustic wave which then travels through medium and it can be characterised through wavelength (m), frequency (Hz), intensity (W/m^2^) and velocity (m/s). Although another acoustic parameter to consider is acoustic impedance, which measures a material’s resistance to the propagation of an acoustic wave. Based on the operating frequency, acoustic waves are classified in four categories, namely, Infrasonic (frequencies less than 20 Hz), Sonic (audible range, 20 Hz to 20 kHz), Ultrasonic (20 kHz up to 200 MHz) and Hypersonic (higher than 200 MHz). Among these, the acoustic waves in ultrasonic range are extensively used in biomedical applications as well as nano bio-robot actuation due to their non-invasive penetration power with respect to biological tissues and ability to provide controllable forces at nanoscale (Cao et al., 2022). Two different strategies are used for acoustic based actuation of nanorobots, acoustic tweezer propulsion and acoustic motion.

- The acoustic tweezer propulsion technique refers to generation of an acoustic field within a workspace and entrapment of drug particles within the acoustic field. Later, with repositioning of the acoustic field, the drug molecules can be actuation/moved to the targeted site.
- Acoustic Motion: Acoustic waves generate vibrations or bubbles around nano bio-robots, propelling them through the bloodstream or other fluids. This technique provides high penetration and precise localization as discussed in (Cao et al., 2024).
- Acoustic Manipulation: An acoustic wave with frequency ranging from KHz to GHz can manipulate object ranging between nano meters to centimetres (Zhao et al., Yang et al.). In (Xu et al., 2022), discussed about various targeted drug release mechanism through acoustic signals. Cao et al. in (Cao et al., 2024) introduced AcoMan which manipulates nanoclusters in water with five degrees of freedom (5-DoF). The acoustic instrument uses an array of 30 ultrasonic elements operating at 1 MHz. AcoMan has manipulation capability within a working space of 5 × 5 × 4 m, achieving a position error of less than 200 μm. Also, a nominal amount of rotational movement is also done by AcoMan.
- Limitations: The precise generation of the required high amplitude, acoustic signal itself poses a challenge along with the fact that such acoustic waves may damage nearby cells as well as tissues.

##### 4.1.1.4 Light-based actuation

The light based nano bio-robot actuation field utilises mechanisms in the nanoscale environment where light interacts with materials to generate forces and manipulate their behaviour. There are five key mechanisms by which a light based manoeuvre is achieved at nanoscale such as.

- Photocatalysis: Light-sensitive materials like titanium dioxide can generate an exciton which is a bound pair of a conduction band electron and a valence band upon light absorption. These excitons create charge gradients of products that propel it through diffusiophoresis. For example, in Au/TiO2 nanomotors, photocatalysis drives the reduction of H+ to H2, generating a concentration gradient and causing motion.
- Photothermal effects: Light-sensitive materials absorb photons and convert them into heat energy, causing propulsion through thermal gradients. Gold nanoparticles, known for their strong plasmonic resonance and efficient photothermal conversion, are often used for this purpose.
- Photomechanical effects: Soft nanorobots made of light-sensitive polymers like liquid crystal elastomers can deform upon light exposure, leading to changes in shape and ultimately, movement. This is particularly useful for mimicking biological movements, according to one study.
- Optical Tweezers: Focused light beams trap and manoeuvre nano bio-robots. Though limited to shallow tissues, this technique offers high spatial accuracy. In Chen B. et al. (2022), Bin et al. have developed a Titanium Oxide based Nanomotor with Nitrogen doping which responds to a wide range of light spectrum. The authors have studied the penetration of nanoparticles in eye using light. This method is used for tasks like assembling nanostructures and manipulating single particles.
- Phototaxis: Some nanorobots can be designed to move towards or away from a light source, similar to the behavior observed in some microorganisms. This phototactic behavior can be achieved through different mechanisms, such as the shadowing effect (where light is partially blocked, leading to uneven excitation) or by programming surface charges.

Light in the range of UV rays ((200–400 nm)) are used for driving photocatalytic reaction in Titanium oxides, Visible light in the range of 400–700 nm are used for optical tweezers and finally Near-Infrared (700–1,100 nm) light is used for deep tissue penetration and nano bio-robot manipulation. However certain limitations exist for this technology.

- Limitations: The light sensitive materials traditionally require high intensity light but produces less stress for actuation. They are limited by penetration depth as well for visible and UV range.

#### 4.1.2 Endogenous method

The endogenous methods outline the use of internal power source developed by chemical and biological reactions after *in vivo* implantation of the nano bio-robots.

##### 4.1.2.1 Chemical actuation

- Fuel-Driven Propulsion: Chemical reactions using biocompatible fuels like hydrogen peroxide or glucose drive nano bio-robot motion. Enzyme-coated nano bio-robots utilize these reactions to generate thrust. For example, catalase-powered nano bio-robots break down hydrogen peroxide into water and oxygen, propelling the robot forward. ATPase can also be used for nano bio-robot propulsion as discussed in Kong et al. (2023).
- Gradient-Based Movement: Chemical gradients, such as pH or ionic concentration, guide nano bio-robots toward target tissues like tumors, exploiting the unique microenvironment (TME) of diseased cells (de Visser and Joyce, 2023). These TME cells are structured cells with both cancer cells and non-malignant cells as well.

##### 4.1.2.2 Microorganism-propelled

Different biological organisms such as bacteria, sperm, etc. are mobile in nature and therefore can act as engines to propel nano bio-robots for drug delivery. This method is still under research as discussed in Chen et al. (2015). When it comes to enhancing robotics and investigating biology, biohybrid strategies that involve living cells, biological tissues, microbes, and even complete animals offer a wide range of opportunities that are not available elsewhere (Ricotti et al., 2017; Mestre et al., 2021).

## 5 Drug release in nano bio-robots

Once nano bio-robots reach their intended target, controlled drug release is critical to maximize therapeutic efficacy and minimize off-target effects. Various mechanisms have been developed to achieve precise and timely release of drugs:

### 5.1 Stimulus-responsive release

Nano bio-robots respond to specific internal or external stimuli to release their payload:

- pH-Triggered Release: Tumors or inflamed tissues often exhibit acidic microenvironments. Nano bio-robots with pH-sensitive coatings such as Eudragit L, S, and F (Poly (methacrylic acid-co-methyl methacrylate)), HPMCP (Hydroxypropyl Methylcellulose phthalate) release drugs only in these conditions.
- Temperature-Sensitive Materials: Heat-responsive nano bio-robots release drugs in response to localized hyperthermia, which can be induced by external lasers or focused ultrasound.

### 5.2 Electric and magnetic field-induced release

- Electric Fields: Applying a mild direct current to nano bio-robots induces changes in their structure or triggers the opening of molecular gates, enabling controlled drug release.
- Magnetic Field-Driven Gates: Magnetic fields control molecular gates or deformable structures on nano bio-robots, allowing precise timing and dosage of drug release as discussed in Liu et al. (2019).

The use of silica nanoparticles (MSNs) equipped with responsive molecular gates can effectively enable controlled release drug delivery. Karimi et al. (2016) discussed the ways in which these regulators can be stimulated by factors (like redox reactions enzymes and pH levels) or external factors (such as light ultrasound magnetic fields) offering a precise way to manage drug release (Karimi et al., 2016).

### 5.3 Mechanical release

- Nano bio-robots with piezoelectric materials release drugs upon mechanical deformation. In Li et al. (2024), a review has been done on the release of targeted drug using combination of piezo material, excited by using an alternating magnetic field. This method can also be triggered externally using ultrasound or vibrations.

### 5.4 Diffusion and polymer degradation

- Diffusion-Controlled Release: Drugs encapsulated within porous nano bio-robots gradually diffuse out over time, regulated by pore size and material properties.
- Biodegradable Polymers: Nano bio-robots constructed from biodegradable materials release drugs as the materials break down naturally within the body.

Nano bio-robots made from polymethyl acrylate (PMA) materials perform controlled drug release through the processes of diffusion as well as polymer relaxation. The research of Corsaro et al. (2021) led to the development of Ag nanoparticles (NPs) within PMA matrices that enhanced drug release according to Fick’s law for nanofibers while demonstrating non-Fickian behavior in the colloidal phase (Corsaro et al., 2021). Hybrid nanoparticles combining polymers and dendrimers are capable of enabling step, by step drug delivery processes. Zhao et al. (2017) on the hand mentioned a nanotechnology approach where paclitaxel (PT) followed by a steady release of doxorubicin (DOXR) is swiftly released. Thus improving the effectiveness of treating multiple drug delivery systems (Zhao et al., 2017).

### 5.5 Release kinetics

The use of nano bio-robots in delivering medications is a method in the field of medicine that provides greater accuracy and management of treatment procedures. Understanding the timing of drug release in nano bio-robots is crucial for maximizing their effectiveness. Guarantee the desired treatment outcomes, nanorobots are engineered to be active, motile, and stimuli-responsive platforms (Zhang et al., 2022). Their drug release behavior is not merely a consequence of passive diffusion or dissolution; it is actively modulated by their propulsion systems, targeting capabilities, and on-demand release triggers (Wells et al., 2019). This means that the kinetic behavior of a nanorobot is not solely determined by the properties of its payload but also by controllable, external parameters, such as the frequency of a magnetic field or the intensity of an ultrasound beam (Liu et al., 2025). Through exploring designs and functions of nano bio-robots researchers are improving the ability to administer medications with greater precision and efficiency (Table 3). A notable progress, in drug delivery methods involves the creation of enzyme powered nanobots that use reactions to improve drug release processes. Hortelao and colleagues conducted a research study in 2018 showing that nanobots fueled by enzymes can boost drug delivery by four times within 6 h compared to inactive versions of the same bots. The increased drug release leads to effectiveness against cancer cells like HeLa cells by leveraging the combined effects of the drug and enzyme powered nanobots (Hortelão et al., 2018).

**TABLE 3:** Drug Release Mechanisms and Kinetics in Various Nano bio-robot-Based Drug Delivery Systems.

Type of Nano bio-robot	Release Mechanism	Release Kinetics	Release Kinetics Model	References
Urease-powered nanobots with a mesoporous silica core-shell structure functionalized with urease enzymes	Enzyme-triggered release via self-propulsion and increased diffusion caused by urease catalysis of urea, leading to enhanced drug release	Drug release increases with urea concentration, showing a four-fold increase in drug release after 6 h compared to passive nanoparticles. Drug release follows enzyme kinetics, stabilizing at higher urea concentrations	Follows Henri–Michaelis–Menten kinetics, indicating enzyme-driven catalytic reaction influencing drug release rate	Hortelão et al. (2018)
Multi-nano bio-robot system using bacterial swarm behavior	Controlled by a Bacterial Swarm Algorithm (BSA) that regulates nano bio-robot population at the target site and adjusts drug molecular weight via quorum sensing	Drug concentration is actively controlled based on AI (autoinducer) signaling and chemotactic responses, preventing excessive accumulation	Algorithm-driven drug release model, adjusting molecular weight and nano bio-robot retention based on AI concentration	Yang et al. (2018)
DNA-based nano bio-robot using aptamers as cargo carriers	Drug release occurs via receptor-mediated endocytosis, where aptamer-drug conjugates bind to cell surface receptors and are internalized into target cells	Drug release is controlled by cellular internalization and receptor binding efficiency, ensuring targeted drug delivery	Follows a stimuli-responsive model, where drug release is triggered by receptor-mediated endocytosis and endosomal escape mechanisms	Citartan et al. (2019)
Polymeric matrix-based drug delivery system (NaCMC)	Drug release occurs through transient diffusion after irradiation, modifying the drug delivery properties	Controlled by diffusion-based mechanisms influenced by polymeric matrix properties	Diffusion-controlled release model, where drug transfer is governed by transient diffusion processes	Sebert et al. (1994)
Magnetic nanoparticle-based nano bio-robot	Drug release is facilitated through thermodynamically driven self-assembly and targeted recognition of malignant cells using fuzzy shape-based identification	Controlled drug delivery via magnetic navigation and targeted interaction with affected cells, ensuring precision drug release at tumor sites	Cybernetic control model, integrating automation and information processing for adaptive drug delivery	Karan and Majumder (2011)
Biohybrid magnetic microrobot using Spirulina microalgae coated with Fe_3_O_4_	Drug release occurs via biodegradation of the microalgae structure, which is tailored by Fe_3_O_4_ coating thickness	Controlled degradation of the Fe_3_O_4_-coated structure allows gradual drug release, ensuring targeted therapy	Biodegradation-driven release model, where drug release is influenced by the thickness of the Fe_3_O_4_ coating	Yan et al. (2017)
Magnetic Resonance (MR)-navigable nano bio-robotic carriers loaded with magnetic nanoparticles (MNPs)	Drug release occurs via localized hyperthermia, which temporarily disrupts the blood-brain barrier (BBB) to allow targeted drug diffusion into brain tissue	Controlled by thermal stimulation, where drug release is activated at the BBB site upon application of an external alternating magnetic field (AC field)	Thermal-stimuli-responsive release model, where drug release is regulated by hyperthermia-induced permeability changes in the BBB.	Tabatabaei et al. (2012)
Atomic Force Microscopy (AFM)-based nano bio-robot with multi-layer coated end-effector	Drug release occurs via gradual diffusion from a multi-layer protein coating on the AFM tip, allowing controlled long-term drug administration	Extended release over ∼40 h, enabling prolonged stimulation of cellular responses	Layer-by-layer diffusion model, where drug release is dictated by the thickness and composition of the coating	Yu et al. (2016)

**TABLE 4:** Nano bio-robotic Strategies for Non-Invasive Delivery.

Delivery Route	Primary Physiological Barriers	Key Nano bio-robotic Strategies	Example	References
Oral	Gastric pH, Enzymatic degradation, Mucus-bicarbonate barrier	Dual-engine propulsion, Biomimicry (macrophage “piggybacking”)	Twin-Bioengine Nano bio-robot (TBY-robot)	Genomics and Rogers (2023)
Nasal	Mucociliary clearance, Blood-brain barrier (BBB)	Enzymatic barrier disruption, Non-invasive nose-to-brain pathway	Catalase-powered nanobots	Serra-Casablancas et al. (2024)
Transdermal	Stratum corneum, Passive diffusion	Active penetration (e.g., electroporation), Microneedle-assisted delivery	DNA-coded nanobots for responsive skincare	Fariha (2025)
Ocular	Vitreous body, Rapid clearance mechanisms	External magnetic field navigation, Ultrasound-triggered release	Hybrid Biomembrane-Functionalized Nano bio-robot (HBFN) for RVO	Wang et al. (2025)

In cancer treatment, Karan and Majumder (2011) designed magnetic nanoparticle based nano bio-robots for targeted drug delivery. Initial work has been carried out on the design of the control system for the nano bio-robot using thermodynamically driven self-assembly and fuzzy shapebased recognition for identification of the malignancy. The release mechanism is based on the target – nano bio-robot interaction, where the drugs are released during the recognition of the cancer cells. The release kinetics are cybernetic control model based, meaning that the release is adaptive and controlled by the environmental signals. This study presents a precision nano bio-robotic system for automated targeted therapy and may represent the potential advancement in the area of intelligent drug delivery and cancer treatment (Karan and Majumder, 2011). Additionally, an inventive method employs nano bio-robot systems managed by algorithms, like the Bacterial Swarm Algorithm (BSA) as proposed by Yang et al. In a study from 2018 mentioned that these systems have the ability to regulate drug release by using quorum sensing and controlling weight levels effectively. By adjusting the quantity of nano bio-robots at the designated location this approach offers management of drug levels which enhances targeted drug delivery (Yang et al., 2018).

To achieve targeted drug delivery and imaging guided therapy, Yan et al. (2017) created biohybrid magnetite microrobots. Microrobots were created by coating Spirulina microalgae with Fe_3_O_4_ for magnetic navigation and imaging, and biodegradability was achieved. The drug release is controlled by the degradation driven model in which the release rate is determined by the thickness of the Fe_3_O_4_ coating. The drug is slowly released in biological environments as the microalgae degrade. This paper describes a novel micro robotic drug delivery system that can navigate precisely, control the release of drugs, and image in real time, thus showing potential for minimal invasive therapeutic applications (Yan et al., 2017).

To develop MR-navigable nano bio-robotic carriers with magnetic nanoparticles (MNPs) for targeted brain drug delivery, Tabatabaei et al. (2012) designed them. Hyperthermia, localized to an area and induced by an alternating magnetic field, opens the blood-brain barrier (BBB) to allow drug diffusion. The release kinetics are controlled by thermal stimulation, thereby ensuring that the drug is delivered precisely to the target and without adverse effects via the systemic circulation. The study further confirms a direct correlation between hyperthermia and the permeability of the BBB and that these effects are reversible. This thermal-stimuli-responsive model offers a promising strategy to achieve non-invasive, controlled drug release for brain-targeted therapies with MRI tracking and magnetic navigation (Tabatabaei et al., 2012).

In a study Sebert et al. (1994) delved into the kinetics of drug release, from irradiated sodium carboxymethyl cellulose (NaCMC). Their research showed that drug movement increased in types of medications after exposure to radiation illustrating the way radiation can improve the release of drugs from these structures in order to achieve controlled drug delivery, Yu et al. (2016) have proposed an AFM based nano bio-robot with a multi-layer coated end effector. The nano bio-robot uses layer by layer diffusion to deliver the drug with the potential of sustaining the release for up to 40 h. The thickness of the coating regulates the release rate, as well as the duration of the therapeutic effect. This system presents a high precision drug delivery system, which can find its application in cellular studies and biomedical sciences. The methodology solves the problems of the previous AFM nano bio-robots, and presents a way to scale up sustained drug release. These mechanisms are important for therapeutic and mechanobiology applications, and this research further develops nano bio-robotic drug delivery systems with potential for long duration target release (Yu et al., 2016).

Aptamers have been investigated for their ability to enhance drug transport by using DNA nanobots. In a study by Citartan and colleagues in 2019 they showed how aptamers can help transport substances such as siRNAs and DNA origami to locations through receptor mediated endocytosis opening up possibilities, for innovative drug delivery systems using DNA nano bio-robots that offer precise management of drug release (Citartan et al., 2019).

## 6 Administration of nano bio-robots

The main drug delivery benefit of nano bio-robots exists through their motile-targeting capability (Zhang et al., 2022). The delivery systems of traditional medicine depend on blood circulation and natural body processes yet nano bio-robots can move independently through biological systems (Zhang et al., 2022). Nano bio-robots use their autonomous movement to bypass biological barriers and travel through complex media thus providing direct access for therapeutic payloads to target sites (Zhang et al., 2022).

The main drawback of passive drug delivery systems involves their low median targeting efficiency which reaches 0.7% (Zhang et al., 2022). Nano bio-robots deliver drugs with high precision which leads to reduced off-target effects and increased therapeutic impact (Singh, 2024). The use of magnetic field-driven motile nanocarriers in preclinical *in vivo* studies demonstrated a 20% improvement in tumor treatment effectiveness when compared to traditional passive nanocarriers (Zhang et al., 2022). The twin-bioengine nano bio-robot demonstrated a 1,000-fold increase in drug concentration at the GI tract diseased site during mouse model studies (Genomics and Rogers, 2023) (Table 4).

The direct delivery of drugs by nano bio-robots enables lower therapeutic agent doses that protect both healthy tissues and reduce systemic side effects (Zhang et al., 2022). The targeted delivery method of nano bio-robots enhances drug effectiveness while protecting patients from adverse effects (Singh, 2024). The trigger-activated release mechanism of payloads from nano bio-robots enhances both drug stability and effectiveness (Singh, 2024). Nano bio-robotic drug delivery systems improve drug bioavailability which stands as a crucial element for therapeutic success (Kumar, 2023).

### 6.1 Oral administration

**FIGURE 5:**
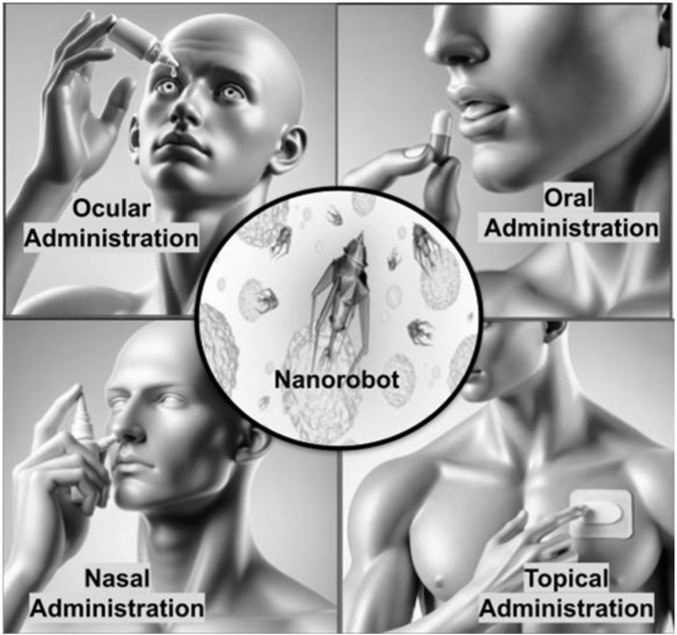
Routes of administration for nano bio-robots.

One of the popular and easy ways to take medication is through oral administration and nano bio-robots are proving to be effective in improving this methods efficiency. The traditional way of delivering drugs orally encounters issues, like breaking down in the GI system and having low bioavailability (Figure 5). However, nano bio-robots can independently travel through the GI tract surpass physiological hurdles and release drugs precisely where they are needed leading to better treatment results. These tiny robots are created to endure the environments in the stomach and intestines so that the payload can reach its target area, with minimal breakdown (Zhang B. et al., 2023).

The TBY-robot demonstrates a formulation that incorporates biodegradable and biocompatible yeast microcapsules as an example (Genomics and Rogers, 2023). The engine contains asymmetrically immobilized enzymes glucose oxidase (GOx) and catalase (Cat) which transform endogenous glucose into a driving force. The enzymatic propulsion system enables the robot to actively break through intestinal mucus which passive systems cannot penetrate (Genomics and Rogers, 2023). The TBY-robots showed effectiveness in mouse models of colitis and gastric ulcers through their multi-modal approach which reduced inflammation and improved disease pathology. The system delivered drugs at a concentration 1,000 times higher than traditional methods at the diseased site which demonstrated its outstanding performance (Genomics and Rogers, 2023). The successful delivery shows how dynamic multi-stage approaches can use body cellular mechanisms to adapt to different physiological areas for targeted delivery.

### 6.2 Nasal administration and inhalation

The nasal delivery route provides CNS access through the blood-brain barrier while reducing systemic side effects by offering a basic non-invasive delivery system (Yoo et al., 2023). The main challenge of this delivery method stems from mucociliary clearance (MCC). The respiratory system depends on mucociliary clearance as its fundamental mechanical defense mechanism to propel mucus and foreign particles through cilia waves toward the pharynx for swallowing or expulsion. The tracheal mucus turnover rate reaches 6–20 mm per minute which forms a significant biological obstacle for drug delivery. Polymer-based nanocarriers that use chitosan as their material find application in intranasal delivery because of their distinctive characteristics (Zhang X. et al., 2023). Chitosan nanoparticles possess strong positive charges which enable them to pass through the BBB (Zhang X. et al., 2023). The human clinical use of polylactic-co-glycolic acid (PLGA) polymers has been approved because their degradation products can be easily eliminated from the body. The drug-loading capacity of these polymer-based systems allows for modification with ligands which improves targeting ability and protects the payload from proteases present in nasal mucus.30 The superior brain drug concentration achieved through intranasal delivery with reduced dosage demonstrates its better targeting ability for CNS applications (Zhang X. et al., 2023). These systems achieve their potential success through their ability to penetrate the mucus barrier actively instead of getting trapped by it.

### 6.3 Transdermal and topical administration

The skin functions as a protective barrier which creates an extremely difficult path for drug delivery (Menichetti et al., 2024). The stratum corneum represents the main obstacle because it consists of a dense hydrophobic layer of dead skin cells embedded in a lipid matrix (Menichetti et al., 2024). The stratum corneum acts as a barrier which prevents most therapeutic agents from passing through except for tiny lipophilic molecules thus making passive diffusion a slow and inefficient process (Menichetti et al., 2024). The dermal tissue contains complex networks of collagen and elastin fibers which create obstacles for drug movement and storage (Alnaim, 2024).

The use of skin patches and creams offer a way to deliver medication without procedures like injections or pills that go through the digestive system. These tiny robots made for this purpose can go through the layer of the skin to reach internal tissues or enter the bloodstream. This approach is beneficial for treating skin issues in areas or for administering drugs in a regulated way. The application of these nano bio-robots, through the skin can provide a release of medication which helps patients follow their treatment plans better and reduces unwanted effects linked to taking medicine orally. Furthermore​. Nano bio-robots utilized in treatments can improve the absorption of medications into the skin​. This makes it a beneficial choice, for dermatological therapies (Das and Sultana, 2024).

Active transdermal therapy can be demonstrated through DNA-coded nanobots which function as skincare agents (Fariha, 2025). The microscopic robots function as real-time skin monitors to detect changes in hydration levels and inflammation and signs of aging. These devices operate by releasing particular active substances when they sense specific changes. The formulation of these nanobots can utilize biocompatible materials such as silk peptides and polyphenols (Fariha, 2025). This technology moves beyond traditional skincare by providing a dynamic, personalized therapeutic response. The most advanced concept includes electro-responsive nanoparticles which can be inserted under the skin to deliver drugs exactly when needed while monitoring drug release rates and sending digital data for distant system adjustments like a tiny programmable IV drip (Fariha, 2025). This technology demonstrates a transition from basic topical use to a self-regulating therapeutic system with closed-loop functionality.

### 6.4 Ocular administration

Delivery of medication to the eyes poses a challenge because of the barriers present in the eye that restrict drug absorption significantly. The delivery of anti-vascular endothelial growth factor (anti-VEGF) agents through intravitreal injections for retinal vein occlusion (RVO) treatment depends on passive diffusion (Wang et al., 2025). The drug delivery process through passive diffusion proves inefficient because the retina receives only restricted drug amounts while the vitreous body’s fast clearance mechanisms reduce drug exposure time. The absence of directional movement represents a major problem which reduces treatment effectiveness and requires multiple non-directed injections (Wang et al., 2025). Nano bio-robots provide an approach by maneuver through these barriers and transporting medications precisely to particular ocular tissues. This method of delivering drugs proves advantageous in managing ailments, like glaucoma, macular degeneration and other retinal disorders. Engineers can design nano bio-robots to dispense drugs in a manner leading to less frequent dosages and improved treatment results. Minimizing exposure and lowering the chances of side effects their skill, in pinpointing particular regions in the eye is notable (Das and Sultana, 2024).

The main approach relies on external magnetic fields to propel nano bio-robots through the vitreous body (Wang et al., 2025). The method enables precise directional movement which surpasses the limitations of passive diffusion to deliver therapeutic agents precisely to the retina. The non-invasive fuel-free propulsion system reduces tissue damage while delivering higher doses of medication to the target area (Wang et al., 2025).

## 7 Pharmacological applications

Medical nano bio-robotics combines nanotechnology with robotics and biomedical engineering to create microscopic systems which perform precise body operations (Sivasankar, 2012). The devices known as Nano Electro Mechanical Systems (NEMS) match the dimensions of biological cells and organelles with diameters below 1 μm (Tang et al., 2024). The devices operate at atomic molecular and cellular levels to overcome traditional medical technique restrictions of non-specific drug distribution and inadequate early diagnosis (Neagu et al., 2024).

### 7.1 Cancer

The most important role of a nano bio-robot in cancer therapy is its ability to target specific areas. The process requires distinguishing cancer cells from normal cells to achieve accurate delivery of therapeutic agents. Researchers have developed multiple nano bio-robot structures to address cancer’s complicated nature (Table 5). DNA Nano bio-robots are designed using DNA origami methods to achieve tumor-specific delivery of payloads by responding to particular molecular indicators (Vimal, 2025). Stimuli-Responsive Nano bio-robots consist of “smart materials” which modify their structure or operational state through environmental signals that occur specifically in cancerous tissues including pH levels temperature and enzymatic activity. Nano bio-robots gain improved navigation and tumor microenvironment interaction through their biomimetic design which replicates urchins spiders and flagellar bacteria (Neagu et al., 2024). The urease-powered nanobot functions as a model intravesical chemotherapy device for bladder cancer treatment (Thomas, 2024).

**TABLE 5:** Nano bio-robot Innovations in Targeted Drug Delivery and Disease Treatment.

Disease Targeted	Nano bio-robot Type	Function	Key Findings	Research Study
Cancer	Magneto nanobots	Drug delivery targeted at tumor sites	Magnetically controlled, pH-sensitive shell, releases drugs directly into cancer cells, reducing side effects. Tested in mice, leading to tumor shrinkage and improved survival	Andhari et al. (2020)
	Magneto-fluorescent nanobots (MFNs)	Isolation of circulating tumor cells (CTCs)	Mg-based self-propelled nanobots capture CTCs efficiently (near 100%) using Fe_3_O_4_ coating and EpCAM-targeting antibodies. Suitable for clinical applications	Wavhale et al. (2021)
	Urease-powered nanomotors	Bladder cancer treatment	Urease enzymatic activity propels nanomotors for enhanced infiltration of bladder cancer spheroids, leading to improved therapeutic agent distribution and potent tumor cell eradication	Simó et al. (2024)
	Bio-inspired nano bio-robots	Cancer treatment and imaging	Designed to mimic natural biological functions for better compatibility and effectiveness in interacting with cancer cells	Nehru et al. (2022)
	Metal-organic framework (MOF) nano bio-robots	Mitochondria-targeting cancer treatment	Programmed to trigger mitochondrial cell death, enhancing cancer treatment efficiency. Verified through laboratory and animal studies	Peng et al. (2023)
	Carbon nano bio-robots	Targeted bio-sensing and photothermal therapy	Designed for cell membrane penetration to enable precise SERS bio-sensing and targeted treatment applications	Chen et al. (2022b)
	ssDNA nano bio-robots	Cancer cell identification and gene suppression	Computationally designed nano bio-robots for cancer cell targeting, gene suppression, and visual examination, demonstrating high stability and effectiveness	Shen et al. (2022)
Cardiovascular Diseases	Magnetic Fe_3_O_4_ Nanoparticles	Thrombosis treatment	rtPA-loaded Fe_3_O_4_ nanoparticles dissolve blood clots efficiently with external magnetic control, reducing rtPA dosage and minimizing bleeding risks. Tested in rabbit carotid artery model	Yang et al. (2023)
Spinal Metastasis	Thrombin-loaded nano bio-robots in hydrogel	Hemostasis and tumor suppression	Injectable hydrogel matrix enables controlled thrombin release for bleeding control in spinal metastasis surgery while also delivering cancer therapy, leading to reduced tumor size. Tested in rabbit metastasis model	Chen et al. (2024)
Diabetes	Self-propelled micromotors	Oral insulin delivery for diabetes management	Micromotors use Mg-stomach acid reaction to propel insulin safely to intestines for absorption, providing a prolonged blood sugar regulation effect for over 5 h	Liu et al. (2023)
	Biohybrid nano bio-robots	Diabetic wound healing	Glycoengineered extracellular vesicle-based nano bio-robots improve tissue penetration, therapeutic delivery, and antioxidant stress management, enhancing wound healing in diabetic mice	Yan et al. (2024)
	AFM-based nano bio-robotic manipulator	Study of β cell mechanics and insulin secretion	Investigates biophysical properties of insulinoma β cells, identifying ion channel function changes linked to glucose-stimulated insulin secretion	Yang et al. (2013)
CNS Injuries	Erythropoietin-based nanobots (ENBs)	CNS injury treatment	Sonication-prepared ENBs provide controlled 24-h EPO release, ensuring sustained neuroprotection and enhanced therapeutic effects for CNS injuries	Le et al. (2022)
Neurological Disorders	Stem cell-based microrobot “Cellbot”	Intranasal stem cell delivery to the brain	Magnetically guided microrobot successfully transported and integrated stem cells into mouse brain tissue, demonstrating potential for treating neurological disorders	Jeon et al. (2021)
	Magnetically operated microrobots	Neural cell transport and connection formation	Microrobots precisely transported neural cells and established functional neural connections, aiding nerve regeneration strategies	Kim et al. (2020)
	Small-scale biorobot	Targeted cell delivery and differentiation	Bioengineered robots successfully transported and differentiated SH-SY5Y cells, supporting potential cell-based therapies for neurological conditions	Jeon et al. (2021)

Nano bio-robots receive functional molecules including aptamers, ligands or antibodies that bind specifically to cancer cell surface markers which are overexpressed (Singh, 2024). A DNA nano bio-robot contains a DNA aptamer which functions as a binding agent for nucleolin because this protein appears only on tumor-associated endothelial cells (Li et al., 2018). The most widely targeted biomarkers in cancer therapy include HER2/neu for breast cancer patients and PSMA for prostate cancer patients (Advances in nano bio-robotics for cancer detection: Targeting 12 types of cancer cells, 2024). Motile-targeting nano bio-robots use active navigation to penetrate tumor tissue which enhances their ability to overcome the limitations that passive drug carriers face when trying to reach the tumor (Zhang et al., 2022). Scientific studies demonstrate that magnetic field-controlled motile nanocarriers enhance tumor treatment outcomes by 20% during *in vivo* studies when compared to conventional passive nanocarriers. The advanced nano bio-robotic systems use programming to operate in synchronized mode as “swarm” behavior (Biswas and Sen, 2016). External controllers direct this coordinated motion to boost tumor penetration and prevent sedimentation and create an 8-fold drug concentration boost at the tumor site (Thomas, 2024). The therapeutic approach delivers maximum treatment effects by minimizing the impact on healthy tissues (Singh, 2024).

After reaching its target destination the nano bio-robot requires mechanisms to perform specific controlled therapeutic functions. Drug payloads exist within nano bio-robots which deploy their contents through specific trigger-based mechanisms. The acidic conditions of tumors can activate pH-sensitive materials to trigger the release of chemotherapy drugs precisely at the tumor site (Singh, 2024). Drug release can be precisely triggered through the use of external laser heat which activates thermal drug delivery (Singh, 2024). The engineers create these nano bio-robots as smart delivery systems for multiple types of agents. DNA nano bio-robots function as drug carriers to deliver thrombin blood coagulation protease to blood vessels within tumors. When thrombin activates coagulation it produces intravascular thrombosis which blocks tumor blood supply and causes necrosis that stops tumor expansion (Vimal, 2025). Nano bio-robots serve as delivery vehicles for both traditional chemotherapy drugs such as doxorubicin and radioactive substances to cancerous cells.11 Radioiodine loaded urease-powered nanobots in mouse experiments reduced bladder tumors by 90% when used at minimal dose levels (Thomas, 2024). The nano bio-robot technology allows heat generation from light exposure for localized cancer cell elimination through hyperthermia (Singh, 2024). Some nano bio-robots operate as “nano surgical blades” through mechanical movements to physically damage tumor cells (Duan, 2024).

The clinical deployment of autonomous nano bio-robots for oncology treatment faces significant obstacles to widespread adoption (Fu et al., 2025). Research on cancer cell lines dominates the field at the *in vitro* level but few studies extend their work to *in vivo* animal models mainly using mice (Neagu et al., 2024). Nano-based cancer therapies encompass passive nanoparticles and diagnostics which have achieved more substantial clinical advancement than “nano-based cancer therapies” as a distinct field (Nanotechnology, 2025). The preclinical data, however, is highly encouraging. The urease-powered nanobots for bladder cancer demonstrate their potential for clinical translation by showing an 8-fold increase in tumor concentration and a 90% tumor shrinkage in mouse models (Thomas, 2024). The DNA nano bio-robot showed both safety and efficacy results in mice and miniature pigs demonstrating its potential as an exact drug delivery system (Li et al., 2018). These devices encounter substantial translation challenges because current manufacturing methods are unsuitable for large-scale clinical use as they remain classified as “laboratory utensils” (Gorrepati et al., 2024). The implementation of these devices in humans requires thorough examination of their long-term biocompatibility together with their body clearance mechanisms (Neagu et al., 2024).

### 7.2 Cardiovascular disease

The field of nanomedicine advances quickly to address cardiovascular diseases through innovative treatments for atherosclerosis as well as myocardial infarction and vascular occlusion (Table 5). Nano bio-robots are currently under development for dual-purpose application in medical diagnostics and precise therapeutic interventions (Nano bio-robotics for health, 2020).

This field incorporates various designed nano bio-robots which serve different functions. The “Trojan Horse” nanoparticles consist of single-walled carbon nanotubes that carry a chemical inhibitor to activate macrophage clearance of cellular debris (Terry, 2020). Small blood vessel thrombectomy and recanalization becomes possible through spiral robots which follow external magnetic field control (Sun et al., 2024). Specialized nano bio-robots utilize iron oxide nanoparticles embedded in hybrid biomembranes to steer magnetic treatments for retinal vein occlusion (RVO) (Wang et al., 2025). A two-stage system was developed to treat myocardial infarction as a treatment method. The device includes a dissolvable base attached to a micro-needle patch. Micro-robots inside needles perform exosome delivery autonomously while the base delivers VEGF-laden nanoparticles (Wang et al., 2025). The creation of stem-cell-based heart patches requires engineers to embed gold nanowires into tissue scaffolds to enhance electrical conductivity. The improvement of heart cell communication through this method leads to a smooth and continuous heart beat.

The advancement of nano bio-robotics technology for cardiovascular disease treatment demonstrates an essential difference between systemic *versus* localized therapeutic methods. The autonomous navigation of plaque-eating nanoparticles through the circulatory system creates difficulties regarding their distribution and elimination from the body. The MI patch along with conductive heart patches function as surgically-implanted localized systems which differ from devices that operate autonomously in the bloodstream. The main obstacles in this method relate to surgical risks and the long-term compatibility of implanted materials instead of the previous navigation and biodistribution issues. These innovative methods will need distinct technical and regulatory pathways because they operate in different ways.

The delivery method for nano bio-robots in retinal vein occlusion treatment demonstrates better performance than traditional medical approaches. The delivery of drugs through intravitreal injections depends on passive diffusion which limits the amount of drug that reaches the retina (Wang et al., 2025). The magnetically-driven nano bio-robots achieve active penetration of the vitreous humor through directional movement to deliver drugs with higher precision and efficacy compared to standard treatments (Wang et al., 2025).

The majority of cardiovascular nano bio-robotics research exists in preclinical development stages where promising results have been achieved in animal testing. Research indicates that plaque-eating nanoparticles are now undergoing testing in large animal models along with human tissue evaluations (Terry, 2020). The MI micro-robot patch underwent successful verification procedures through thoracoscopic surgery on rabbits and pigs (Wang et al., 2025). The concept of heart patches containing gold nanowires remains experimental because it requires animal testing for validation. The translation of these devices faces significant hurdles because implant-based devices like conductive heart patches require long-term biocompatibility testing to prevent immune reactions and implant-related heart complications.

### 7.3 Diabetes

The global number of adults with diabetes stands at 537 million but experts predict this figure will increase to 783 million by 2045 (Lemmerman et al., 2020). The standard treatment approaches of insulin therapy and oral medications encounter difficulties with patient adherence and unwanted medication reactions. People with Type 1 diabetes need constant tracking and exact insulin administration for their diabetes management. Nano bio-robots show great potential for diabetes treatment through their ability to monitor blood glucose levels in real time and deliver insulin precisely to the body (Cavalcanti et al., 2008b; A and M, 2018). The natural pancreatic function of insulin production can be replicated through nano bio-robotic systems which function as an alternative to current invasive medical approaches (Volpatti et al., 2020).

The fundamental concept of nano bio-robotics for diabetes treatment involves creating a pancreatic “closed-loop” insulin secretion system which patients with diabetes have lost (Volpatti et al., 2020). Glucose oxidase (GOx) functions as the main enzyme in Enzymatic Sensing systems. The nano bio-robots contain glucose oxidase that changes blood glucose to gluconic acid (Anderson and Langer, 2013). The main detection process depends on this enzymatic reaction. The gluconic acid produced by GOx reaction is detected through FETs which represent nanomaterial-based sensors. The gluconic acid conversion process generates acid pH while producing negative charges which the sensor can detect for precise glucose level measurement (Puri, 2024).

These systems distribute insulin according to the output of their sensing mechanisms. The glucose-sensitive nanogels utilize modified dextran polymers which respond to acidic conditions (Anderson and Langer, 2013). High glucose levels that get converted to gluconic acid cause the dextran spheres to dissociate which releases the insulin stored inside (Anderson and Langer, 2013). Researchers achieved insulin release kinetics control by mixing different acetal contents into dextran polymers when developing these systems (Volpatti et al., 2020). The insulin delivery system can generate both immediate insulin bursts and continued sustained insulin release through its designed mechanism (Volpatti et al., 2020).

Nano bio-robotic approaches for diabetes management have demonstrated successful results in preclinical research. An injection of MIT-developed gel into Type 1 diabetic mice maintained healthy blood-sugar levels during a period of 10 days (Anderson and Langer, 2013). The system delivered 16 h of glycemic control to diabetic mice which proved its glucose-responsive functionality in living subjects (Volpatti et al., 2020). The technological advancements have not yet entered human clinical trials. The main obstacle lies in matching the system’s response rate to the fast insulin release speed of natural pancreatic islet cells (Anderson and Langer, 2013). Researchers perform extended studies and work to enhance the system’s dosage levels for human clinical applications (Anderson and Langer, 2013). The protein-based GOx systems encounter stability issues alongside scalability problems and possible immune system reactions (Volpatti et al., 2020).

### 7.4 Neurological disorders

The treatment of central nervous system disorders faces distinctive challenges because the blood-brain barrier (BBB) blocks 98% of therapeutic compounds from penetrating the brain (Singh, 2024). Nano bio-robots are presently being created to break through this biological challenge which enables the therapeutic treatment of neurodegenerative disorders and brain traumas.

The design of nano bio-robots for CNS applications requires specialized features which specifically address the unique difficulties found in neural tissue. Nano bio-robots implement magnetic nanoparticles as carriers to transport live nerve cells (neurons) as well as therapeutic agents. These devices can move through brain tissue under the direction of an external magnetic field (Kim et al., 2020). Scientists developed the “Cell Rover” as a compact flat antenna which can be moved inside cells through magnetic field manipulations (Ogasa, 2023). The devices can be equipped with sensors which enable them to detect particular molecules including Alzheimer’s disease early indicator misfolded proteins (Ogasa, 2023). Nanosized hydrogels unite traditional hydrogel benefits such as biocompatibility and fluid-like transport characteristics with the advantageous small size and enhanced permeability properties of nanoparticles (Hersh et al., 2022). Nano bio-robot systems can be engineered to deliver drugs based on their degradation patterns or through activation by temperature or pH changes (Hersh et al., 2022).

The therapeutic applications of nano bio-robots in the CNS represent a fundamental shift in treatment philosophy. The delivery of functioning neurons through magnetic nano bio-robots enables targeted brain tissue repair of damaged neural pathways (Kim et al., 2020). The approach goes beyond symptom relief because it focuses on restoring damaged neural connections to treat the fundamental cause of pathology (Kim et al., 2020). “Cell Rover” exemplifies this new paradigm. The device enables early neurodegenerative disease detection through misfolded protein detection which serves as an early Alzheimer’s indicator (Ogasa, 2023). Researchers work on creating nano bio-robots to transport dopamine medication for Parkinson’s patients and to extract beta-amyloid plaques from Alzheimer’s patients. The nanogels function as drug delivery vehicles that transport chemotherapy agents to brain tumors before releasing them in the tumor’s acidic microenvironment after crossing the BBB (Hersh et al., 2022). The development of fleets of remote-control nano bio-robots aims to enable microsurgical procedures such as brain tumor detection and destruction while eliminating the requirement for traditional surgical approaches (Ogasa, 2023). Nano bio-robots for the CNS have aspirational objectives which transition from basic symptom treatment to precise cellular healing and tissue growth. The field’s ultimate goal lies in developing “miniaturized surgeons” who will execute precise cellular repairs at the microscopic level. Nano bio-robotics represents a fresh therapeutic concept which works to stop disease evolution by targeting its origin (Kim et al., 2020).

CNS nano bio-robotics research is currently at the proof-of-concept and preclinical development stage (Université de Montréal, 2015). Scientists have shown the successful delivery of neurons to brain tissue slices but this has not been replicated in living organisms (Kim et al., 2020). Nano bio-robots face a main challenge for clinical trial entry because they struggle to navigate the human body particularly in the sensitive complex CNS (Nano bio-robots move closer to clinical trials with new model that helps them navigate through the bloodstream, n. d.).

## 8 Global market

Nano bio-robots receive increasing interest because they have the potential to transform healthcare through direct cell-targeted drug delivery which reduces side effects and boosts treatment effectiveness. Research and development (R&D) investments drive forward this advanced technology which operates in the market space (Li J. et al., 2017).

**FIGURE 6:**
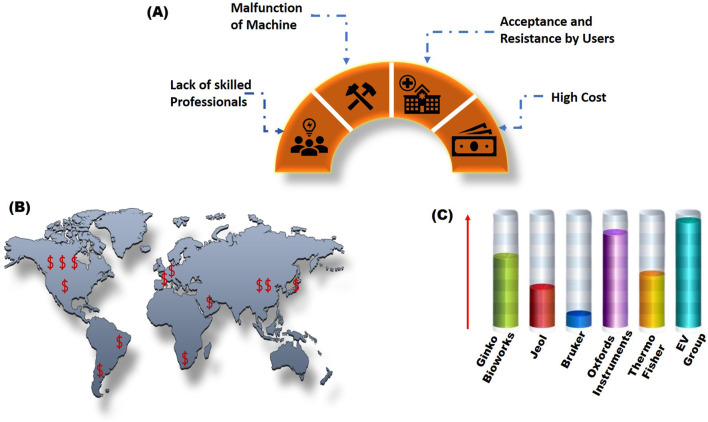
Global market scenario of nano bio-robots manufactured by various multinational companies. **(A,B)** represent the key drivers of nano bio-robots and country's expenditure in the manufacturing of these nano bio-robots in dollars respectively. **(C)** provides a pert chart showing a list of world wide companies involved in production of nano bio-robots.

The nano bio-robots industry remains in its initial development stage at the global level. The market demonstrates substantial potential for growth during the upcoming years (Figure 6). The market research indicates that the nano bio-robots industry started at USD 5 billion in 2021 and analysts expect it to reach USD 25 billion by 2030 2030 (Pokrajac et al., 2021) with a projected growth rate above 20% from 2021 to 2030 (Advisor, 2022). Nanotechnology advancements together with the rising demand for innovative healthcare solutions drive the current market growth (Zhou et al., 2021).

### 8.1 Key market drivers

The nano bio-robots market grows worldwide because of various elements which drive its expansion (Figure 6). The rising healthcare requirements act as the primary element that drives this market trend. The growing population of elderly individuals combined with increasing cases of cancer and diabetes leads to rising demands for precise and efficient medical treatments. Nano bio-robots serve as solutions to healthcare problems by providing medication delivery with high precision (Geiger and Hirschl, 2015).

The nanotechnology field supports market growth through its substantial developments in nanomaterial production techniques and fabrication methods during the previous 10 years. Nano bio-robots now have expanded functionality because of recent technological advances which enable their use in healthcare and environmental monitoring for molecular-level pollutant detection and counteraction (Leenes et al., 2017).

Research and development investments from government entities and private organizations drive market advancement through innovative breakthroughs. Nanotechnology research funding from governments of developed countries is increasing because they understand its potential to drive economic growth and improve public health. The field of nano bio-robotics receives substantial financial support from both established companies and emerging startups which enables them to develop advanced technologies for multiple industry applications (Martineau et al., 2020).

## 9 Challenges and ethical issues

**FIGURE 7:**
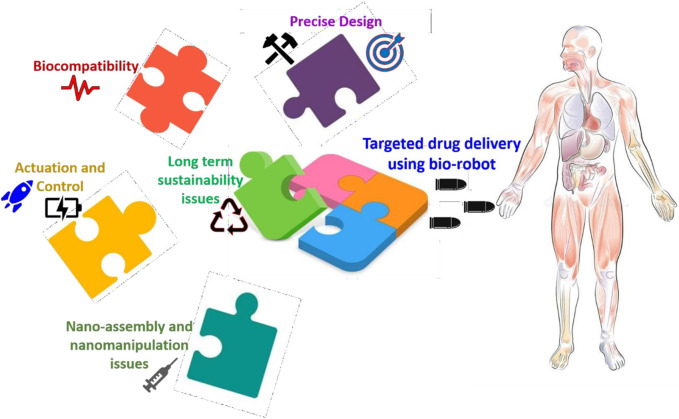
Schematic representation portraying the different challenges in nano bio-robot manufacturing, its actuation mechanism and sustainability.

Machines functioning at the nanoscale shows great potential for transforming diverse industries, from surgical expertise to healthcare intervention. Their capacity to execute duties at that level unveils new opportunities for personalized medicine that were once beyond imagination. However, the incorporation of sophisticated technologies, into medical applications raises crucial moral, ethical issues and regulations. The idea of targeted drug delivery by bio-robot is still proof-of-concept. Grand challenges faced by this system lies with design of the robot, size, fabrication, transportation, control, power supply, cost and biocompatibility issues (Le and Shim, 2024) (Figure 7).

Design of a bio-robot relies on the work environment; temperature gradient and blood flow rate sustaining the main issue while designing the robot for human body application. A key step in designing the robot is to target the disease and organ for which the machine is going to work (Gao et al., 2015). Like a regular robot, bio-robots are also made with a basic frame and parts that can sense and control things. Both organic and inorganic materials (polymers), including DNA and protein, can be used to create these components. However, emerging manufacturing techniques (VHDL: Very High Speed Integrated Circuit Hardware Description Language) as well as new developments in transducers, computation, and manipulation in the Integrated circuit made possible the development of précised bio-robots (Jiang et al., 2005; Cavalcanti et al., 2008b). Further in the locomotion, propulsion and control stages, bio-robot faces many challenges regarding the external stimuli type and its amount (Peyer et al., 2013; Nguyen et al., 2019). Among the various external stimuli, magnetic fields are currently the most widely studied and evaluated for *in vivo* applications as it offered noninvasive, fuel free safe platform (less than 3 Tesla) for human body. Similarly, acoustic fields also been employed in medical diagnostics due to their established clinical use but it faces depth penetration limitations (Shim et al., 2019).

Another important aspect that needs to be properly taken into account while developing bio-robotic systems is biocompatibility. After entering the body, the possibility of triggering inflammatory reactions has to be taken into account. Using live materials (bacteria, etc.) accidental harm outside the intended location can happen which can trigger the unnecessary difficulties (Martel et al., 2004). Therefore, thorough toxicity assessments are crucial for such biologically active systems, concentrating on long-term safety and clinical application in addition to small animal model data. Li et al. (2012) vividly described the relevance of toxicity using a system-based approach focusing on lung, dermal, liver, and nervous system targets that are well discussed (Li et al., 2012).

Though bio-robot faces multiple challenges, we have to always admits that creating treatments that surpass current ones is the primary goal of bio-robot development. Thus, the design of drug-delivery techniques, particularly loading and release techniques, should be the main emphasis of future study.

One major ethical dilemma arises when new surgical technologies like nano bio-robots are being introduced in life medical environments as discussed in the study “Innovation in surgical technology and techniques”. The integration of healthcare technologies comes with various uncertainties such as concerns about their safety and effectiveness and the need, for appropriate regulatory measures to safeguard patient wellbeing (Geiger and Hirschl, 2015). The rapid advancement of nano bio-robotics technology creates additional concerns because authorities may struggle to monitor the fast-evolving technology which might produce gaps in oversight. The ethical challenges of nano bio-robots stem directly from the problems they generate. The document “Guidelines for Dealing with Legal and Ethical Concerns in Regulating Robotics Technologies examines the complex process of overseeing advanced robotics technologies including nano bio-robots. The writers observe that existing legal systems fail to address the problems these technologies create between protecting fundamental rights and advancing technology. Nano bio-robots require special attention to regulation because their medical and sensitive field applications increase the importance of this issue (Leenes et al., 2017).

Maintaining research integrity is essential to ensure that the advancement of nano bio-robots follows guidelines with transparency and accountability at its core. The process involves testing and validation of nano bio-robots before their implementation, in clinical settings and continuous monitoring to identify and resolve any ethical concerns that may emerge. Organizational ethics focuses on ensuring that entities engaged in nano bio-robot development prioritize safety and welfare above financial gains.

Relational ethics underlines the significance of including patients and the general public in the decision-making process concerning the advancement of nano bio-robots technology development. It is crucial that patients are not educated about the possible advantages and risks but also have a say in shaping the discussions regarding the utilization of these technologies due to their potential impact. By involving patients in these conversations regarding nano bio-robot deployment strategies can aid in establishing trust among developers and healthcare providers while ensuring that their use aligns, with societal norms and expectations.

## 10 Conclusion

The development of drug transport bio-robotics systems marks a major advancement toward better targeted therapeutic approaches. Researchers have created innovative drug delivery methods through the application of micro and nanoscale robotics principles to overcome conventional drug delivery limitations. These systems demonstrate the ability to explore biological complexities while delivering therapeutic agents precisely to target cells and tissues which leads to promising disease treatment possibilities for cancer and neurological disorders and infectious diseases. Animal model preclinical research has proven both the practicality and effectiveness of different bio-robot designs which now move toward clinical implementation.

The field continues to encounter major obstacles despite its remarkable progress. The development of bio-robotics requires sustained research to solve essential challenges including material biocompatibility and biodegradability testing and body navigation precision and scalable manufacturing and immune system response management. The advancement of bio-robotics technology for drug delivery will be influenced by three main factors including improved autonomous systems and artificial intelligence integration with advanced imaging methods and the development of new materials and propulsion systems.

The successful clinic implementation of bio-robotics depends on technological progress and solutions for regulatory issues and economic considerations and societal factors related to these technologies. The widespread adoption of bio-robotics for patient benefit worldwide will require establishing clear regulations and demonstrating economic value while engaging public discussions to resolve ethical concerns. The complete realization of bio-robotics potential for medical revolution depends on sustained interdisciplinary teamwork between researchers and engineers and clinicians and policymakers.

## Abbreviations

1D/ 2D/ 3D/4D, One–Dimensional/ Two–Dimensional/ Three–Dimensional/ Four–Dimensional; CVD, Chemical Vapor Deposition; AI, Artificial Intelligence; NDDS, nano biorobotic drug-delivery system; DNA, Deoxyribonucleic Acid; EBL, Electron-beam lithography; FIB, Focused ion beam; MBE, Molecular Beam Epitaxy; TPP, Two-Photon Polymerization; miRNA, MicroRNA; Fe_x_O_x_, Iron Oxides; MNR, Magnetic nanorobots; DoF, Degrees of freedom.

## References

[B1] Advisor N. O. (2022). Nanobots market size, share and analysis report, 2022-2030. Available online at: https://www.novaoneadvisor.com/report/nanobots-market (Accessed August 24, 2025).

[B2] Ahmed S. Amin S. E. Elarif T. (2015). “Navigation and cooperative control for nanorobots in the bloodstream environment based on swarm intelligence,” in Volume 9: 2015 ASME/IEEE international conference on mechatronic and embedded systems and applications (American Society of Mechanical Engineers). 10.1115/detc2015-46506

[B3] Allen T. M. Cullis P. R. (2013). Liposomal drug delivery systems: from concept to clinical applications. Adv. Drug Deliv. Rev. 65, 36–48. 10.1016/j.addr.2012.09.037 23036225

[B4] Alnaim A. S. (2024). Nanocrystals in dermal drug delivery: a breakthrough for enhanced skin penetration and targeted skin disorder treatments. Pharmaceutics 16, 1561. 10.3390/pharmaceutics16121561 39771540 PMC11676127

[B5] Amadi E. V. Venkataraman A. Papadopoulos C. (2022). Nanoscale self-assembly: concepts, applications and challenges. Nanotechnology 33, 132001. 10.1088/1361-6528/ac3f54 34874297

[B6] An M. Feng Y. Liu Y. Yang H. (2023). External power-driven micro/nanorobots: design, fabrication, and functionalization for tumor diagnosis and therapy. Prog. Mat. Sci. 140, 101204. 10.1016/j.pmatsci.2023.101204

[B7] Andhari S. S. Wavhale R. D. Dhobale K. D. Tawade B. V. Chate G. P. Patil Y. N. (2020). Self-propelling targeted magneto-nanobots for deep tumor penetration and pH-responsive intracellular drug delivery. Sci. Rep. 10, 4703. 10.1038/s41598-020-61586-y 32170128 PMC7070039

[B146] Anderson D. Langer R. (2013). Nanotechnology could help fight diabetes. MIT News | Massachusetts Institute of Technology. Available online at: https://news.mit.edu/2013/nanotechnology-could-help-fight-diabetes-0516 .

[B8] Asahi H. Horikoshi Y. (2019). Molecular beam epitaxy (Nashville, TN: John Wiley and Sons).

[B10] Benouhiba A. Wurtz L. Rauch J.-Y. Agnus J. Rabenorosoa K. Clévy C. (2021). NanoRobotic structures with embedded actuation via ion induced folding. Adv. Mat. 33, e2103371. 10.1002/adma.202103371 34554607

[B11] Biswas O. Sen A. (2016). “Nanorobot—The expected ever reliable future asset in diagnosis, treatment, and therapy,” in Foundations and frontiers in computer, communication and electrical engineering (CRC Press), 451–456. 10.1201/b20012-88

[B12] Buchnev O. Grant-Jacob J. A. Eason R. W. Zheludev N. I. Mills B. MacDonald K. F. (2022). Deep-learning-assisted focused ion beam nanofabrication. Nano Lett. 22, 2734–2739. 10.1021/acs.nanolett.1c04604 35324209 PMC9097578

[B13] Cao H. X. Jung D. Lee H.-S. Nguyen V. D. Choi E. Kang B. (2022). Holographic acoustic tweezers for 5-DoF manipulation of nanocarrier clusters toward targeted drug delivery. Pharmaceutics 14, 1490. 10.3390/pharmaceutics14071490 35890382 PMC9317593

[B14] Cao H. Wang C. Shen C. (2024). Review of the applications of Micro/Nano bio-robots in Biomedicine. ACS Appl. Nano Mater. 24. 10.3390/pharmaceutics14071490

[B15] Castillo-Henríquez L. Vargas-Zúñiga R. Pacheco-Molina J. Vega-Baudrit J. (2020). Electrospun nanofibers: a nanotechnological approach for drug delivery and dissolution optimization in poorly water-soluble drugs. ADMET DMPK 8, 325–353. 10.5599/admet.844 35300196 PMC8915594

[B16] Cavalcanti A. (2023). Carbon nanotubes for nanorobotics: expanding the possibilities. J. Nanosci. Curr. Res. 8, 1–2. 10.37421/2572-0813.2023.8.194

[B17] Cavalcanti A. Shirinzadeh B. Freitas R. A. Jr Hogg T. (2008a). Nanorobot architecture for medical target identification. Nanotechnology 19, 015103. 10.1088/0957-4484/19/01/015103

[B18] Cavalcanti A. Shirinzadeh B. Kretly L. C. (2008b). Medical nanorobotics for diabetes control. Nanomedicine 4, 127–138. 10.1016/j.nano.2008.03.001 18455965

[B19] Chen K. Chen T. Liu H. Yang Z. (2015). “A Pt/Au hybrid self-actuating nanorobot towards to durg delivery system,” in 10th IEEE international conference on Nano/Micro engineered and molecular systems (IEEE), 286–289. 10.1109/nems.2015.7147428

[B20] Chen X. Dai S. Parmar J. Martínez-Villacorta A. M. Gómez-Vallejo V. Llop J. (2018). Micro and nanorobots in biomedical applications: from bench to bedside. ACS Nano 12, 1220–1227. 10.1021/acsnano.7b07220 29361216

[B21] Chen M. Lee H. Yang J. Xu Z. Huang N. Chan B. P. (2020). Electrohydrodynamic 3D nanoprinting: parallel, multi‐material electrohydrodynamic 3D nanoprinting (small 13/2020). Small 16, 2070070. 10.1002/smll.202070070 32101385

[B22] Chen B. Ding M. Tan H. Wang S. Liu L. Wang F. (2022a). Visible-light-driven TiO2@N-Au nanorobot penetrating the vitreous. Appl. Mat. Today 27, 101455. 10.1016/j.apmt.2022.101455

[B23] Chen Y. Pan R. Wang Y. Guo P. Liu X. Ji F. (2022b). Carbon helical nanorobots capable of cell membrane penetration for single cell targeted SERS bio‐sensing and photothermal cancer therapy. Adv. Funct. Mat. 32, 2200600. 10.1002/adfm.202200600

[B24] Chen Q. Yan M. Hu A. Liang B. Lu H. Zhou L. (2024). Injectable nanorobot-hydrogel superstructure for hemostasis and anticancer therapy of spinal metastasis. Nanomicro Lett. 16, 259. 10.1007/s40820-024-01469-3 39085736 PMC11291792

[B25] Citartan M. Kaur H. Presela R. Tang T.-H. (2019). Aptamers as the chaperones (Aptachaperones) of drugs-from siRNAs to DNA nanorobots. Int. J. Pharm. 567, 118483. 10.1016/j.ijpharm.2019.118483 31260780

[B26] Corsaro C. Neri G. Mezzasalma A. M. Fazio E. (2021). Weibull modeling of controlled drug release from Ag-PMA nanosystems. Polym. (Basel) 13, 2897. 10.3390/polym13172897 34502937 PMC8434431

[B27] Das T. Sultana S. (2024). Multifaceted applications of micro/nanorobots in pharmaceutical drug delivery systems: a comprehensive review. Futur. J. Pharm. Sci. 10, 2. 10.1186/s43094-023-00577-y

[B28] De Visser K. E. Joyce J. A. (2023). The evolving tumor microenvironment: from cancer initiation to metastatic outgrowth. Cancer Cell 41, 374–403. 10.1016/j.ccell.2023.02.016 36917948

[B29] Drexler E. (1987). Engines of creation: the coming era of nanotechnology. Anchor.

[B30] Dreyfus R. Baudry J. Roper M. L. Fermigier M. Stone H. A. Bibette J. (2005). Microscopic artificial swimmers. Nature 437, 862–865. 10.1038/nature04090 16208366

[B31] Duan Y. (2024). Applications of nanorobots in targeted therapy for cancer. Highlights Sci. Eng. Technol. 120, 592–601. 10.54097/z22k4n32

[B32] Escorcia-Díaz D. García-Mora S. Rendón-Castrillón L. Ramírez-Carmona M. Ocampo-López C. (2023). Advancements in nanoparticle deposition techniques for diverse substrates: a review. Nanomater. (Basel) 13, 2586. 10.3390/nano13182586 37764615 PMC10537803

[B33] Fariha F. T. (2025). “DNA-Coded skincare with nanobots,” in TexSPACE today. Available online at: https://www.texspacetoday.com/dna-coded-skincare-with-nanobots/ (Accessed August 18, 2025).

[B34] Feynman R. (2012). “There’s plenty of room at the bottom,” in Electrical engineering handbook. CRC Press, 3–12. 10.1201/b11930-3

[B35] Fleischer R. (1966). “Fantastic voyage,” in 20th century Fox Film Corporation.

[B36] Nebraska Center for Materials & Nanoscience (2012). Focused Ion Beam. Available online at: https://nanofab.unl.edu/fib/ (Accessed August 22, 2025).

[B37] Fu B. Luo D. Li C. Feng Y. Liang W. (2025). Advances in micro-/nanorobots for cancer diagnosis and treatment: propulsion mechanisms, early detection, and cancer therapy. Front. Chem. 13, 1537917. 10.3389/fchem.2025.1537917 39981265 PMC11839623

[B38] Gao W. Dong R. Thamphiwatana S. Li J. Gao W. Zhang L. (2015). Artificial micromotors in the mouse’s stomach: a step toward *in vivo* use of synthetic motors. ACS Nano 9, 117–123. 10.1021/nn507097k 25549040 PMC4310033

[B39] Geiger J. D. Hirschl R. B. (2015). Innovation in surgical technology and techniques: challenges and ethical issues. Semin. Pediatr. Surg. 24, 115–121. 10.1053/j.sempedsurg.2015.02.008 25976146

[B40] Genomics F. L. Rogers M. (2023). Nanorobots that navigate to inflamed sites: a new method of drug delivery? Front. Line Genomics. Available online at: https://frontlinegenomics.com/nanorobots-that-navigate-to-inflamed-sites-a-new-method-of-drug-delivery/ (Accessed August 18, 2025).

[B41] Gorrepati N. Quazi F. Mohammed PhD A. S. Avacharmal R. (2024). Use of nanorobots in Neuro chemotherapy diagnosis in human. Int. J. Glob. Innovations Solutions (IJGIS). 10.21428/e90189c8.7a880e58

[B143] Hersh A. M. Alomari S. Tyler B. M. (2012). Crossing the blood-brain barrier: Advances in nanoparticle technology for drug delivery in neuro-oncology. Int. J. Mol. Sci. 23, 4153. 10.3390/ijms23084153 35456971 PMC9032478

[B42] Högberg B. Hammarskjöld A. (2024). Nanorobot with hidden weapon kills cancer cells. Karolinska Institutet. Available online at: https://news.ki.se/nanorobot-with-hidden-weapon-kills-cancer-cells (Accessed August 22, 2025).

[B43] Hortelão A. C. Patiño T. Perez-Jiménez A. Blanco À. Sánchez S. (2018). Enzyme-powered nanobots enhance anticancer drug delivery. Adv. Funct. Mat. 28, 1705086. 10.1002/adfm.201705086

[B44] Hu Y. (2018). College of chemistry, chemical engineering and biotechnology, Donghua University. in State key laboratory for modification of chemical fibers and polymer materials

[B45] Hu M. Ge X. Chen X. Mao W. Qian X. Yuan W.-E. (2020). Micro/nanorobot: a promising targeted drug delivery system. Pharmaceutics 12, 665. 10.3390/pharmaceutics12070665 32679772 PMC7407549

[B46] Huang Z. Chi-Pong Tsui G. Deng Y. Tang C.-Y. (2020). Two-photon polymerization nanolithography technology for fabrication of stimulus-responsive micro/nano-structures for biomedical applications. Nanotechnol. Rev. 9, 1118–1136. 10.1515/ntrev-2020-0073

[B47] Hulla J. E. Sahu S. C. Hayes A. W. (2015). Nanotechnology: history and future. Hum. Exp. Toxicol. 34, 1318–1321. 10.1177/0960327115603588 26614822

[B48] Jeon S. Park S. H. Kim E. Kim J.-Y. Kim S. W. Choi H. (2021). A magnetically powered stem cell‐based microrobot for minimally invasive stem cell delivery *via* the intranasal pathway in a mouse brain (adv. Healthcare mater. 19/2021). Adv. Healthc. Mat. 10, 2170089. 10.1002/adhm.202170089 34160909

[B49] Jiang H. W. Wang S. G. Xu W. Zhang Z. Z. He L. (2005). “Construction of medical nano bio-robot,” in *Proceedings of the 2005 IEEE International Conference on Robotics and Biomimetics-ROBIO*, (Hong Kong, China), 151–154.

[B50] Ju X. Chen C. Oral C. M. Sevim S. Golestanian R. Sun M. (2025). Technology roadmap of micro/nanorobots. ACS Nano 19, 24174–24334. 10.1021/acsnano.5c03911 40577644 PMC12269370

[B51] Kandasamy G. Maity D. (2015). Recent advances in superparamagnetic iron oxide nanoparticles (SPIONs) for *in vitro* and *in vivo* cancer nanotheranostics. Int. J. Pharm. 496, 191–218. 10.1016/j.ijpharm.2015.10.058 26520409

[B52] Kandasamy G. Sudame A. Luthra T. Saini K. Maity D. (2018). Functionalized hydrophilic superparamagnetic iron oxide nanoparticles for magnetic fluid hyperthermia application in liver cancer treatment. ACS Omega 3, 3991–4005. 10.1021/acsomega.8b00207 30023884 PMC6044893

[B53] Karan S. Majumder D. D. (2011). “Molecular machinery-a nanorobotics control system design for cancer drug delivery,” in 2011 international conference on recent trends in information systems (IEEE). 10.1109/retis.2011.6146867

[B54] Karimi M. Mirshekari H. Aliakbari M. Sahandi-Zangabad P. Hamblin M. R. (2016). Smart mesoporous silica nanoparticles for controlled-release drug delivery. Nanotechnol. Rev. 5. 10.1515/ntrev-2015-0057

[B55] Karimi K. Fardoost A. Mhatre N. Rajan J. Boisvert D. Javanmard M. (2024). A thorough review of emerging technologies in micro- and nanochannel fabrication: limitations, applications, and comparison. Micromachines (Basel) 15, 1274. 10.3390/mi15101274 39459148 PMC11509582

[B56] Kim E. Jeon S. An H.-K. Kianpour M. Yu S.-W. Kim J.-Y. (2020). A magnetically actuated microrobot for targeted neural cell delivery and selective connection of neural networks. Sci. Adv. 6, eabb5696. 10.1126/sciadv.abb5696 32978164 PMC7518876

[B57] Klocke V. Gesang T. (2003). “Nanorobotics for micro production technology,” in Fiber-based component fabrication, testing, and connectorization. 10.1117/12.469152

[B58] Kong X. Gao P. Wang J. Fang Y. Hwang K. C. (2023). Advances of medical nanorobots for future cancer treatments. J. Hematol. Oncol. 16, 74. 10.1186/s13045-023-01463-z 37452423 PMC10347767

[B59] Kumar M. (2023). Nanorobotics based drug delivery system: recent developments and future prospects. IJCRT 11, 630–640.

[B60] Le Q.-V. Shim G. (2024). Biorobotic drug delivery for biomedical applications. Molecules 29, 3663. 10.3390/molecules29153663 39125066 PMC11314275

[B61] Le T. H. Nguyen C. T. Koo K.-I. Hwang C. H. (2022). Erythropoietin nanobots: their feasibility for the controlled release of erythropoietin and their neuroprotective bioequivalence in central nervous system injury. Appl. Sci. (Basel) 12, 3351. 10.3390/app12073351

[B62] Lee K. Y. Mooney D. J. (2001). Hydrogels for tissue engineering. Chem. Rev. 101, 1869–1880. 10.1021/cr000108x 11710233

[B63] Lee S. (2002). NEMS: the heart of next-gen actuators. Number Anal. LLC. Available online at: https://www.numberanalytics.com/blog/nems-the-heart-of-next-gen-actuators (Accessed August 22, 2025).

[B64] Leenes R. Palmerini E. Koops B.-J. Bertolini A. Salvini P. Lucivero F. (2017). Regulatory challenges of robotics: some guidelines for addressing legal and ethical issues. Law Innov. Technol. 9, 1–44. 10.1080/17579961.2017.1304921

[B65] Lemmerman L. R. Das D. Higuita-Castro N. Mirmira R. G. Gallego-Perez D. (2020). Nanomedicine-based strategies for diabetes: diagnostics, monitoring, and treatment. Trends Endocrinol. Metab. 31, 448–458. 10.1016/j.tem.2020.02.001 32396845 PMC7987328

[B66] Li X. Wang L. Fan Y. Feng Q. Cui F.-Z. (2012). Biocompatibility and toxicity of nanoparticles and nanotubes. J. Nanomater. 2012, 548389–19. 10.1155/2012/548389

[B67] Li T. Li J. Zhang H. Chang X. Song W. Hu Y. (2016). Magnetically propelled fish-like nanoswimmers. Small 12, 6098–6105. 10.1002/smll.201601846 27600373

[B68] Li J. Esteban-Fernández de Ávila B. Gao W. Zhang L. Wang J. (2017a). Micro/nanorobots for biomedicine: delivery, surgery, sensing, and detoxification. Sci. Robot. 2, eaam6431. 10.1126/scirobotics.aam6431 31552379 PMC6759331

[B69] Li T. Li J. Morozov K. I. Wu Z. Xu T. Rozen I. (2017b). Highly efficient freestyle magnetic nanoswimmer. Nano Lett. 17, 5092–5098. 10.1021/acs.nanolett.7b02383 28677387

[B70] Li S. Jiang Q. Liu S. Zhang Y. Tian Y. Song C. (2018). A DNA nanorobot functions as a cancer therapeutic in response to a molecular trigger *in vivo* . Nat. Biotechnol. 36, 258–264. 10.1038/nbt.4071 29431737

[B71] Li A. Yang J. He Y. Wen J. Jiang X. (2024). Advancing piezoelectric 2D nanomaterials for applications in drug delivery systems and therapeutic approaches. Nanoscale Horiz. 9, 365–383. 10.1039/d3nh00578j 38230559

[B72] Liu J. F. Jang B. Issadore D. Tsourkas A. (2019). Use of magnetic fields and nanoparticles to trigger drug release and improve tumor targeting. Wiley Interdiscip. Rev. Nanomed. Nanobiotechnol. 11, e1571. 10.1002/wnan.1571 31241251 PMC6788948

[B73] Liu K. Liu Q. Yang J. Xie C. Wang S. Tong F. (2023). Micromotor based mini-tablet for oral delivery of insulin. ACS Nano 17, 300–311. 10.1021/acsnano.2c07953 36546656

[B74] Liu X. Xu Z. Che Y. Guo Z. Jin D. Wang Q. (2025). Mechanical agitation-assisted transmembrane drug delivery by magnetically powered spiky nanorobots. Res. (Wash. D.C.) 8, 0768. 10.34133/research.0768 40809455 PMC12349924

[B75] Lombardo D. Calandra P. Pasqua L. Magazù S. (2020). Self-assembly of organic nanomaterials and biomaterials: the bottom-up approach for functional nanostructures formation and advanced applications. Mater. (Basel) 13, 1048. 10.3390/ma13051048 32110877 PMC7084717

[B76] Lu X. Liu J. Wu X. Ding B. (2019). Multifunctional DNA origami nanoplatforms for drug delivery. Chem. Asian J. 14, 2193–2202. 10.1002/asia.201900574 31125182

[B9] Mukhopadhyay A Prosenjit M. (2018). Application of nano-biotechnology for improvement in therapeutic approaches for the treatment of diabetes. *J. Clin. Mol. Endocrinol.*03 03. 10.21767/2572-5432.100047

[B77] Martel S. (2010). Collective methods of propulsion and steering for untethered microscale nanorobots navigating in the human vascular network. Proc. Inst. Mech. Eng. Part C 224, 1505–1513. 10.1243/09544062jmes2079

[B78] Martel S. (2014). Magnetic therapeutic delivery using navigable agents. Ther. Deliv. 5, 189–204. 10.4155/tde.13.147 24483196

[B79] Martel S. Mathieu J. B. Felfoul O. Macicior H. Beaudoin G. Soulez G. (2004). Adapting MRI systems to propel and guide microdevices in the human blood circulatory system. Conf. Proc. IEEE Eng. Med. Biol. Soc. 2004, 1044–1047. 10.1109/IEMBS.2004.1403342 17271861

[B80] Martel S. Felfoul O. Mohammadi M. Mathieu J.-B. (2008). Interventional procedure based on nanorobots propelled and steered by flagellated magnetotactic bacteria for direct targeting of tumors in the human body. Annu. Int. Conf. IEEE Eng. Med. Biol. Soc. 2008, 2497–2500. 10.1109/IEMBS.2008.4649707 19163210

[B81] Martineau J. T. Minyaoui A. Boivin A. (2020). Partnering with patients in healthcare research: a scoping review of ethical issues, challenges, and recommendations for practice. BMC Med. Ethics 21, 34. 10.1186/s12910-020-0460-0 32393230 PMC7216517

[B82] McCray W. P. (2007). MBE deserves a place in the history books. Nat. Nanotechnol. 2, 259–261. 10.1038/nnano.2007.121 18654274

[B83] Menichetti A. Mordini D. Montalti M. (2024). Penetration of microplastics and nanoparticles through skin: effects of size, shape, and surface chemistry. J. Xenobiot. 15, 6. 10.3390/jox15010006 39846538 PMC11755607

[B84] Mestre R. Patiño T. Sánchez S. Guix M. Fuentes J. Valerio-Santiago M. (2021). 3D-bioengineered model of human skeletal muscle tissue with phenotypic features of aging for drug testing purposes. Wiley Interdiscip. Rev. Nanomed. Nanobiotechnol 13. 10.1088/1758-5090/ac165b 34284359

[B87] Neagu A.-N. Jayaweera T. Weraduwage K. Darie C. C. (2024). A nanorobotics-based approach of breast cancer in the nanotechnology era. Int. J. Mol. Sci. 25, 4981. 10.3390/ijms25094981 38732200 PMC11084175

[B88] Nehru S. Misra R. Bhaswant M. (2022). Multifaceted engineered biomimetic nanorobots toward cancer management. ACS Biomater. Sci. Eng. 8, 444–459. 10.1021/acsbiomaterials.1c01352 35118865

[B89] Nelson B. J. Kaliakatsos I. K. Abbott J. J. (2010). Microrobots for minimally invasive medicine. Annu. Rev. Biomed. Eng. 12, 55–85. 10.1146/annurev-bioeng-010510-103409 20415589

[B90] Nguyen J. Z. Go K. T. Kang G. Min B. Kim H. K. Kim S. J. (2019). Multifunctional nanorobot System for active therapeutic delivery and synergistic chemo-photothermal therapy. Nano Lett. 19, 8550–8564. 10.1021/acs.nanolett.9b03051 31694378

[B142] Ogasa N. (2023). Nanobots can now enter brain cells to spy on what they’re doing. Science News Explores. Available online at: https://www.snexplores.org/article/nanobots-can-now-enter-brain-cells-to-spy-on-what-theyre-doing (Accessed August 18, 2025).

[B91] Park H. Otte A. Park K. (2022). Evolution of drug delivery systems: from 1950 to 2020 and beyond. J. Control. Release 342, 53–65. 10.1016/j.jconrel.2021.12.030 34971694 PMC8840987

[B92] Peng X. Tang S. Tang D. Zhou D. Li Y. Chen Q. (2023). Autonomous metal-organic framework nanorobots for active mitochondria-targeted cancer therapy. Sci. Adv. 9, eadh1736. 10.1126/sciadv.adh1736 37294758 PMC10256165

[B93] People O. (2023). Electrospinning. Nanosci. Instrum. Available online at: https://www.nanoscience.com/techniques/electrospinning/ (Accessed August 22, 2025).

[B94] Peyer K. E. Zhang L. Nelson B. J. (2013). Bio-inspired magnetic swimming microrobots for biomedical applications. Nanoscale 5, 1259–1272. 10.1039/c2nr32554c 23165991

[B95] Pokrajac L. Abbas A. Chrzanowski W. Dias G. M. Eggleton B. J. Maguire S. (2021). Nanotechnology for a sustainable future: addressing global challenges with the international Network4Sustainable nanotechnology. ACS Nano 15, 18608–18623. 10.1021/acsnano.1c10919 34910476

[B141] Puri A. (2024). Nanosensor technologies for improved glucose monitoring. AZoNano. Available online at: https://www.azonano.com/article.aspx?ArticleID=6692 (Accessed August 18, 2025).

[B96] Prizes N. (2020). Overview. The University of Sheffield. Available online at: https://sheffield.ac.uk/ebl/overview (Accessed August 22, 2025).

[B144] Ramachandran R. V. Bhat R. Saini D. K. Ghosh A. (2021). Theragnostic nanomotors: Successes and upcoming challenges. Wiley Interdiscip Rev. Nanomed. Nanobiotechnol. 13(6), e1736. 10.1002/wnan.1736 34173342

[B97] Ricotti L. Trimmer B. Feinberg A. W. Raman R. Parker K. K. Bashir R. (2017). Biohybrid actuators for robotics: a review of devices actuated by living cells. Sci. Robot. 2, eaaq0495. 10.1126/scirobotics.aaq0495 33157905

[B98] Sebert P. Bourny E. Rollet M. (1994). Gamma irradiation of carboxymethylcellulose: technological and pharmaceutical aspects. Int. J. Pharm. 106, 103–108. 10.1016/0378-5173(94)90307-7

[B99] Serra-Casablancas M. Di Carlo V. Esporrín-Ubieto D. Prado-Morales C. Bakenecker A. C. Sánchez S. (2024). Catalase-powered nanobots for overcoming the mucus barrier. ACS Nano 18, 16701–16714. 10.1021/acsnano.4c01760 38885185 PMC11223492

[B100] Sharma E. Rathi R. Misharwal J. Sinhmar B. Kumari S. Dalal J. (2022). Evolution in lithography techniques: microlithography to nanolithography. Nanomater. (Basel) 12, 2754. 10.3390/nano12162754 36014619 PMC9414268

[B101] Shen X. Ouyang Q. Tan H. Ouyang J. Na N. (2022). Computationally designed ssDNA modular nanorobots for cancer recognition, toehold disintegration, visual diagnosis and synergistic gene silencing. Res. Square. 10.21203/rs.3.rs-1026581/v1 36999978

[B102] Shim G. Le Q.-V. Suh J. Choi S. Kim G. Choi H.-G. (2019). Sequential activation of anticancer therapy triggered by tumor microenvironment-selective imaging. J. Control. Release 298, 110–119. 10.1016/j.jconrel.2019.02.012 30771413

[B103] Simó C. Serra-Casablancas M. Hortelao A. C. Di Carlo V. Guallar-Garrido S. Plaza-García S. (2024). Urease-powered nanobots for radionuclide bladder cancer therapy. Nat. Nanotechnol. 19, 554–564. 10.1038/s41565-023-01577-y 38225356 PMC11026160

[B104] Singh A. (2024). Nanorobots: robotic drug delivery systems. AZoRobotics. Available online at: https://www.azorobotics.com/Article.aspx?ArticleID=733 (Accessed August 12, 2025).

[B105] Sitti M. (2009). “Microscale and nanoscale robotics systems Micro-Nanorobotics,” in IEEE Robotics and Automation Magazine, 16, 58–70.

[B106] Sivasankar M. (2012). Brief review on nano robots in bio medical applications. *Adv. Robot. Autom.* 01 01. 10.4172/2168-9695.1000101

[B107] Sun T. Chen J. Zhang J. Zhao Z. Zhao Y. Sun J. (2024). Application of micro/nanorobot in medicine. Front. Bioeng. Biotechnol. 12, 1347312. 10.3389/fbioe.2024.1347312 38333078 PMC10850249

[B108] Tabatabaei S. N. Duchemin S. Girouard H. Martel S. (2012). Towards MR-navigable nanorobotic carriers for drug delivery into the brain. IEEE Int. Conf. Robot. Autom., 727–732. 10.1109/ICRA.2012.6225041 23518572 PMC3601978

[B109] Tang D. Peng X. Wu S. Tang S. (2024). Autonomous nanorobots as miniaturized surgeons for intracellular applications. Nanomater. (Basel) 14, 595. 10.3390/nano14070595 38607129 PMC11013175

[B110] Thomas L. (2024). Nanobots for bladder cancer treatment, promising high efficacy and targeted delivery. News-Medical. Available online at: https://www.news-medical.net/news/20240115/Nanobots-for-bladder-cancer-treatment-promising-high-efficacy-and-targeted-delivery.aspx (Accessed August 12, 2025).

[B140] Terry M. (2020). Researchers develop nanoparticles to eat plaques that cause heart attacks and strokes. BioSpace. Available online at: https://www.biospace.com/nanoparticles-that-eat-away-the-plaques-that-cause-heart-attacks (Accessed August 18, 2025).

[B111] Tsuda S. (2016). “Nanorobotics,” in Encyclopedia of nanotechnology (Dordrecht: Springer Netherlands), 2641–2645. 10.1007/978-94-017-9780-1_137

[B145] Université de Montréal (2015). Nanorobotic agents open the blood-brain barrier, offering hope for new brain treatments. Science Daily. Available online at: https://www.sciencedaily.com/releases/2015/03/150325101942.htm (Accessed August 18, 2025).

[B112] Vadlamani L. I. (2010). Nanobots as therapeutic devices. SSRN Electron. J. 10.2139/ssrn.1693838

[B113] Vasantha Ramachandran R. Bhat R. Kumar Saini D. Ghosh A. (2021). Theragnostic nanomotors: successes and upcoming challenges. Wiley Interdiscip. Rev. Nanomed. Nanobiotechnol. 13, e1736. 10.1002/wnan.1736 34173342

[B114] Vimal J. (2025). Nanobots in the healthcare - applications, benefit, and key challenges. DelveInsight Bus. Res. Available online at: https://www.delveinsight.com/blog/nanobots-in-the-healthcare-sector (Accessed August 12, 2025).

[B139] Volpatti L. R. Matranga M. A. Cortinas A. B. Delcassian D. Daniel K. B. Langer R. (2020). Glucose-responsive nanoparticles for rapid and extended self-regulated insulin delivery. ACS Nano 14, 488–497. 10.1021/acsnano.9b06395 31765558

[B115] Wang G. (2018). Nanotechnology: the new features. arXiv [cs.ET]. Available online at: http://arxiv.org/abs/1812.04939.

[B116] Wang B. Kostarelos K. Nelson B. J. Zhang L. (2021). Trends in micro-/nanorobotics: materials development, actuation, localization, and system integration for biomedical applications. Adv. Mat. 33, e2002047. 10.1002/adma.202002047 33617105

[B117] Wang S. Chen X. Liu Y. Jiang Y. Li J. Ren L. (2025). Hybrid biomembrane-functionalized nanorobots penetrate the vitreous body of the eye for the treatment of retinal vein occlusion. ACS Nano 19, 7728–7741. 10.1021/acsnano.4c12327 39964811

[B118] Wavhale R. D. Dhobale K. D. Rahane C. S. Chate G. P. Tawade B. V. Patil Y. N. (2021). Water-powered self-propelled magnetic nanobot for rapid and highly efficient capture of circulating tumor cells. Commun. Chem. 4, 159. 10.1038/s42004-021-00598-9 36697678 PMC9814645

[B119] Weerarathna I. N. Kumar P. Dzoagbe H. Y. Kiwanuka L. (2025). Advancements in micro/nanorobots in medicine: design, actuation, and transformative application. ACS Omega 10, 5214–5250. 10.1021/acsomega.4c09806 39989765 PMC11840590

[B120] Wegner K. D. Hildebrandt N. (2015). Quantum dots: bright and versatile *in vitro* and *in vivo* fluorescence imaging biosensors. Chem. Soc. Rev. 44, 4792–4834. 10.1039/c4cs00532e 25777768

[B121] Wei F. Zhong T. Zhan Z. Yao L. (2021). Self‐assembled micro‐nanorobots: from assembly mechanisms to applications. ChemNanoMat 7, 238–252. 10.1002/cnma.202000608

[B122] Wells C. M. Harris M. Choi L. Murali V. P. Guerra F. D. Jennings J. A. (2019). Stimuli-responsive drug release from smart polymers. J. Funct. Biomater. 10, 34. 10.3390/jfb10030034 31370252 PMC6787590

[B124] Xu K. Xu S. Wei F. (2021). Recent progress in magnetic applications for micro- and nanorobots. Beilstein J. Nanotechnol. 12, 744–755. 10.3762/bjnano.12.58 34367858 PMC8313977

[B125] Xu Y. Bian Q. Wang R. Gao J. (2022). Micro/nanorobots for precise drug delivery *via* targeted transport and triggered release: a review. Int. J. Pharm. 616, 121551. 10.1016/j.ijpharm.2022.121551 35131352

[B126] Yan X. Zhou Q. Vincent M. Deng Y. Yu J. Xu J. (2017). Multifunctional biohybrid magnetite microrobots for imaging-guided therapy. Sci. Robot. 2, eaaq1155. 10.1126/scirobotics.aaq1155 33157904

[B127] Yan C. Feng K. Bao B. Chen J. Xu X. Jiang G. (2024). Biohybrid nanorobots carrying glycoengineered extracellular vesicles promote diabetic wound repair through dual-enhanced cell and tissue penetration. Adv. Sci. (Weinh.) 11, e2404456. 10.1002/advs.202404456 38894569 PMC11336935

[B128] Yang R. Xi N. Lai K. W. C. Patterson K. C. Chen H. Song B. (2013). Cellular biophysical dynamics and ion channel activities detected by AFM-based nanorobotic manipulator in insulinoma β-cells. Nanomedicine 9, 636–645. 10.1016/j.nano.2012.10.011 23178285 PMC3594338

[B129] Yang H. Li M. Cao J. He D. (2018). “A bacterial swarm algorithm to control drug release by multi-nanorobots,” in 2018 IEEE international conference on Real-time Computing and Robotics (RCAR) (IEEE). 10.1109/rcar.2018.8621716

[B130] Yang P. Ren J. Yang L. (2023). Nanoparticles in the New Era of cardiovascular therapeutics: challenges and opportunities. Int. J. Mol. Sci. 24, 5205. 10.3390/ijms24065205 36982284 PMC10049183

[B131] Yoo J. Tang S. Gao W. (2023). Micro- and nanorobots for biomedical applications in the brain. Nat. Rev. Bioeng. 1, 308–310. 10.1038/s44222-023-00038-4

[B132] Yu J. Yang Y. Seiffert-Sinha K. Lee I. Xi N. Sinha A. A. (2016). “Multi-layer coated nanorobot end-effector for efficient drug delivery,” in 2016 IEEE 16th international conference on nanotechnology (IEEE-NANO). 10.1109/nano.2016.7751526

[B133] Zhang D. Liu S. Guan J. Mou F. (2022). “Motile-targeting” drug delivery platforms based on micro/nanorobots for tumor therapy. Front. Bioeng. Biotechnol. 10, 1002171. 10.3389/fbioe.2022.1002171 36185435 PMC9523273

[B134] Zhang B. Zhu L. Pan H. Cai L. (2023a). Biocompatible smart micro/nanorobots for active gastrointestinal tract drug delivery. Expert Opin. Drug Deliv. 20, 1427–1441. 10.1080/17425247.2023.2270915 37840310

[B135] Zhang X. Wang M. Liu Z. Wang Y. Chen L. Guo J. (2023b). Transnasal-brain delivery of nanomedicines for neurodegenerative diseases. Front. Drug Deliv. 3, 1247162. 10.3389/fddev.2023.1247162 40838047 PMC12363324

[B136] Zhao Z. Lou S. Hu Y. Zhu J. Zhang C. (2017). A nano-in-nano polymer-dendrimer nanoparticle-based nanosystem for controlled multidrug delivery. Mol. Pharm. 14, 2697–2710. 10.1021/acs.molpharmaceut.7b00219 28704056

[B137] Zhao Q. Cheng N. Sun X. Yan L. Li W. (2023). The application of nanomedicine in clinical settings. Front. Bioeng. Biotechnol. 11, 1219054. 10.3389/fbioe.2023.1219054 37441195 PMC10335748

[B138] Zhou H. Mayorga-Martinez C. C. Pané S. Zhang L. Pumera M. (2021). Magnetically driven micro and nanorobots. Chem. Rev. 121, 4999–5041. 10.1021/acs.chemrev.0c01234 33787235 PMC8154323

